# Supplementary material for: Body mass index and all-cause mortality in a 21st century U.S. population: A National Health Interview Survey analysis
Source: PLoS One. 2023 Jul 5;18(7):e0287218. doi: 10.1371/journal.pone.0287218 (PMC10321632; doi:10.1371/journal.pone.0287218)
Supplement: S1 Appendix — (DOCX) [file pone.0287218.s001.docx]

**S1 Appendix. Extended Supplementary Methods**

**NHIS Dataset**

The National Health Interview Survey (NHIS) is a nationally representative survey of the civilian, noninstitutionalized United States population. The National Center for Health Statistics (NCHS) publicly releases surveys annually. These surveys can then be concatenated across multiple years using NCHS guidelines (National Center for Health Statistics Survey Description and National Health Interview Survey, 2016).^13^ We pooled surveys from 1999 through 2018 to create a final NHIS 1999-2018 dataset. From 1999 to 2018, the sample followed a multi-stage, probability design that involved geographically based sampling units, along with methods to partition the target universe into clusters and strata and oversample minority sub-populations (non-Hispanic Black, non-Hispanic Asian, Hispanic groups).^13^ While the survey design has largely remained consistent across the years included, there have been changes in number of participants sampled, number of geographic sampling units used, the oversampling strategy (e.g. oversampling of Asians and elderly individuals starting in 2006), and variables included. Further information about the dataset and the sample design can be found elsewhere (Parsons, 2014).^14^ The final unconditional response rates for the “Sample Adult” component of NHIS ranged from 69.6% in 1999 to 58.9% in 2014 (National Center for Health Statistics, 1999, 2014)^14^, with a progressive decrease over time. Because this dataset was publicly available, de-identified, and population-based, this study was exempt from Rutgers Institutional Review Board approval.

**Inclusion and Exclusion Criteria**

A total of 554,332 non-pregnant adults ≥20 years old with recorded BMI were included. We excluded participants with missing data on education (N=1445, 0.35% of sample), insurance coverage (N=1279, 0.34% of sample), and citizenship status (N=680, 0.14% of sample). We also excluded individuals with a BMI greater than 99 or less than 10 as these BMIs are generally outliers or unfeasible measurements. For those with uninterpretable data (e.g. refusal, not ascertained, not sure) on comorbidities, we assumed they did not have the condition and coded it as “No”.

**Nuances of Exposure Measurement**

BMI: Since NHIS 1997, men's heights were top-coded to 76 inches and women's to 70 inches, while weights were top-coded to 299 Ibs in men and 274 lbs in women. These were done to reduce inaccuracies in self-reported height and weight.^14^

**NHANES Dataset: Sensitivity Analysis**

NHANES is a nationally representative survey of the civilian, non-institutionalized United States population. The survey, implemented by the National Center for Health Statistics (NCHS), follows a complex, stratified, multistage probability design consisting of interviews, physical examinations at home or at a mobile examination center, and laboratory testing. Further information about the dataset and the sample design can be found elsewhere.^17^ We included individuals with data on measured BMI and covariates. Data from NHANES was also linked to death certificates from the National Death Index (NDI) as of December 31st, 2019, allowing for mortality analysis. We used identical inclusion and exclusion criteria as NHIS. Due to smaller sample size (N=44,308), we collapsed several BMI categories and used the following categories: <22.5, 22.5-24.9, 25.0-27.4, 27.5-29.9, 30.0-34.9, ≥35. We then assessed BMI-mortality associations in multiple subgroups, including high (≥102 cm for men, ≥88 cm for women) vs. normal waist circumference, self-reported weight change in the past year (unintentional weight loss of ≥10 pound, intentional weight loss or less than 10 pound weight change, ≥10 pound weight gain), and disease status (presence of any one of the following: self-reported COPD, emphysema, chronic bronchitis, non-skin cancer, current liver disease, cardiovascular disease (CAD, HF, stroke, MI), chronic kidney disease (eGFR<60 per CKD-EPI equation), other kidney disease, asthma, chronic prescription medication use (chronic CNS agents, chronic respiratory agents, anticoagulation, cancer agents), moderate to severe anemia (Hgb <10 g/dl), malnutrition (<3.2 g/dl serum albumin), unintentional weight loss of >10 pounds in the past year). In the main analysis, we did not adjust for comorbidities as they are likely on the causal pathway or potential colliders. However, in a sensitivity analysis to explore the independent association between BMI and mortality, we adjusted for metabolic syndrome criteria (waist circumference, triglycerides, fasting glucose, systolic blood pressure, HDL-c). We also utilized self-reported maximum lifetime BMI (calculated using measured current height and self-reported maximum weight), which several researchers have utilized recently to reduce the bias associated with weight loss over time from occult disease.^18-20^ For maximum lifetime BMI, we used age at maximum BMI and age at death or censoring as the time scales to determine time-to-event.

Covariates:

We selected covariates a priori based on prior studies and clinical knowledge. Demographic variables included age, sex, race/ethnicity (determined by respondents' primary reported race [variable name MRACBPI2], categorized into non-Hispanic White, non-Hispanic Black, non-Hispanic Asian, Hispanic, and Other [American Indian, Alaskan Native, Multiracial, Other]), education level (graduate level or higher degree, some college/Bachelor's degree, high school graduate or lower), citizenship status (U.S. citizen, Non-U.S. citizen), number of years in the U.S. (Native born, Immigrated: 0–5, 5–10, 10–15, ≥15 years), region of residence (Northeast, Midwest, South, West), and insurance status (has coverage, no coverage). Sociobehavioral factors included marital status (married, never married, separated/divorced/widowed, and other), smoking status (ever smoker, non-smoker), alcohol consumption (ever had a drink, never had a drink), physical activity (sufficient, insufficient; sufficient physical activity as determined by U.S. guidelines (Piercy et al., 2018): ≥30 min of moderate-level activity on ≥5 days/week, ≥25 min of vigorous-level activity on ≥3 days/week, or any combination of the two), and muscle strengthening activity (≥2, <2 days/week). Comorbidities included self-reported cardiovascular disease (coronary artery disease, stroke, myocardial infarction, other heart disease), history of non-skin cancer or melanoma, COPD, emphysema, or chronic bronchitis, current asthma, liver disease, weak/failing kidneys, hypertension diagnosis, diabetes diagnosis (no specification of Type I vs. II; gestational diabetes not included as pregnant women excluded from study), and presence of depressive symptoms based on the Kessler-6 scale, a well-validated tool to measure depression. Finally, healthcare/clinic factors included any doctor's visits in the past 12 months (yes/no) and mental health appointments (yes/no). Questionnaire protocols and quality control procedures for each covariate are described elsewhere.^13^
