# Supplementary material for: Body mass index and all-cause mortality in a 21st century U.S. population: A National Health Interview Survey analysis
Source: PLoS One. 2023 Jul 5;18(7):e0287218. doi: 10.1371/journal.pone.0287218 (PMC10321632; doi:10.1371/journal.pone.0287218)
Supplement: S2 Appendix — (DOCX) [file pone.0287218.s002.docx]

**S2 Appendix. Supplementary File**

**Supplementary Figure S1.** Directed Acyclic Graph (DAG) Diagram for BMI and All-cause Mortality

Legend: This figure is a crude representation of our proposed DAG for the association between BMI and all-cause mortality. We can assume BMI is causally associated with diabetes and diabetes is causally associated with mortality. If there is an unmeasured confounder, such as poor nutrition, that is associated with BMI, and is causally associated with diabetes and mortality, diabetes becomes a collider (BMI and poor nutrition ‘collide’ on diabetes). Adjusting for diabetes but not for diet (as it is unmeasured) may create a spurious association between BMI and diet and affect the association between BMI and mortality through this pathway. Preston and Stokes explain this phenomenon in much greater detail and more eloquently in the following paper: Preston SH, Stokes A. Obesity paradox: conditioning on disease enhances biases in estimating the mortality risks of obesity. Epidemiology (Cambridge, Mass.). 2014 May;25(3):454.

**Supplementary Figure S2**. BMI Distribution by Subgroups.

Legend: Figure S2 depicts the distribution of BMI, determined via self-reported weight and height and rounded to the nearest integer, across various demographic subgroups within the 1999-2018 NHIS cohort. (A) BMI distribution by age group; (B) BMI distribution by gender; (C) BMI distribution by earliest and latest 4-year combination of survey cycles; (4) BMI distribution by race/ethnicity. Races/ethnicities not included in this figure include American Indian/Alaskan Natives, Multi-racial individuals, and individuals who did not report race.

**Supplementary Table S1.** Crude Five-year and Overall Mortality Rates by Subgroups (NHIS 1999-2018)

**Supplementary Table S2.** Crude Five-year and Overall Mortality Rates by Survey Cycle Year (NHIS 1999-2018)

**Supplementary Figure S3**. Association between BMI and All-cause Mortality by Age Group Among NHIS 1999-2018 Never-smoking Participants

Legend: Figure S3 shows the hazard ratios for BMI categories, relative to a BMI of 22.5-24.9, by age group. Confidence bands represent 95% CI. The blue line represents all individuals. The red line depicts healthy, never-smoking individuals. Healthy defined as no self-reported history of cardiovascular disease or non-skin cancer or melanoma. (A) presents the hazard ratios among individuals aged 20-64 overall. (B) presents the hazard ratios for adults ≥65 years overall. (C) presents the hazard ratios among individuals aged 20-64, excluding individuals who died within 2 years of follow-up. (D) presents the hazard ratios among adults ≥65 years, excluding individuals who died within 2 years of follow-up.

**Supplementary Figure S4.** Association between BMI and All-cause Mortality by Disease Status Among NHIS 1999-2018 Never-smoking Participants

Legend: Figure S4 shows the hazard ratios for BMI categories, relative to a BMI of 22.5-24.9. The red line depicts never-smoking individuals without minor morbidity, defined as self-reported history of cardiovascular disease, non-skin cancer or melanoma, COPD, current asthma, liver disease, kidney disease, diabetes, or functional limitations. The blue line depicts never-smoking participants without major morbidity, defined via Berrington de Gonzalez et al. as self-reported history of cardiovascular disease and non-skin cancer except melanoma.

**Supplementary Table S3.** Baseline Characteristics by BMI Category Among 1999-2018 NHANES Participants

**Supplementary Table S4.** Comparison of Demographic Factors and BMI Across NHANES and NHIS Cohorts

**Supplementary Table S5.** Association between BMI and All-cause Mortality Among NHANES Participants by Age Group

**Supplementary Table S6.** Association between BMI and All-cause Mortality Among NHANES Participants by High Waist Circumference

**Supplementary Table S7.** Association between BMI And All-cause Mortality Among NHANES Participants by Weight Change Status

**Supplementary Table S8.** Association between BMI And All-cause Mortality Among NHANES Participants by Disease Status

**Supplementary Table S9**. Association between Maximum Lifetime BMI and Mortality Among NHANES Participants by Age Group

**Supplementary Table S10**. Association between BMI and Mortality Among NHIS Participants: Exclusion of First Five Years of Follow-up

**Figure S1.** Directed Acyclic Graph (DAG) Diagram for BMI and All-cause Mortality


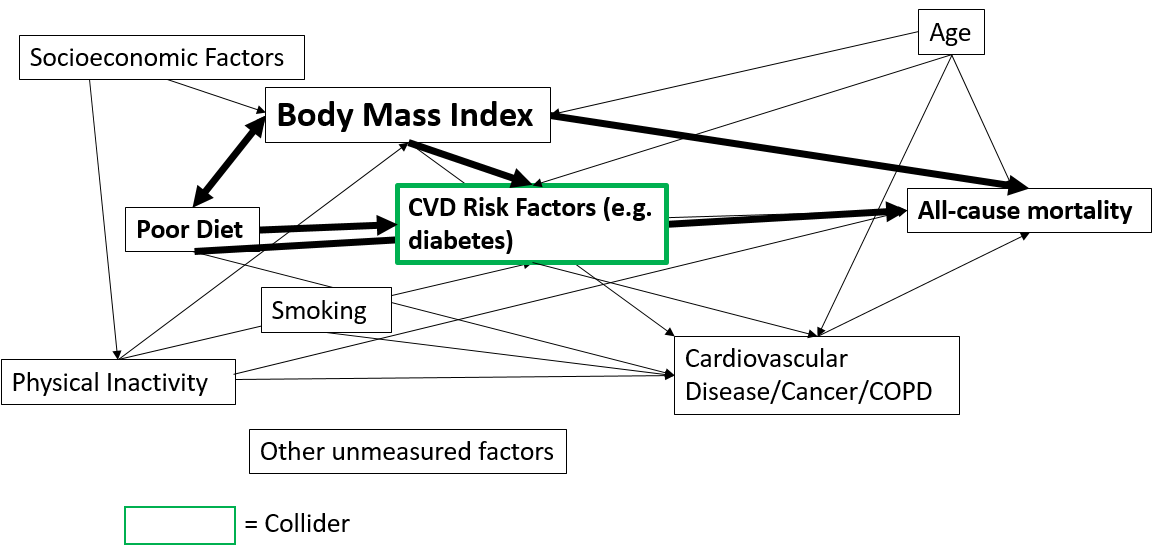


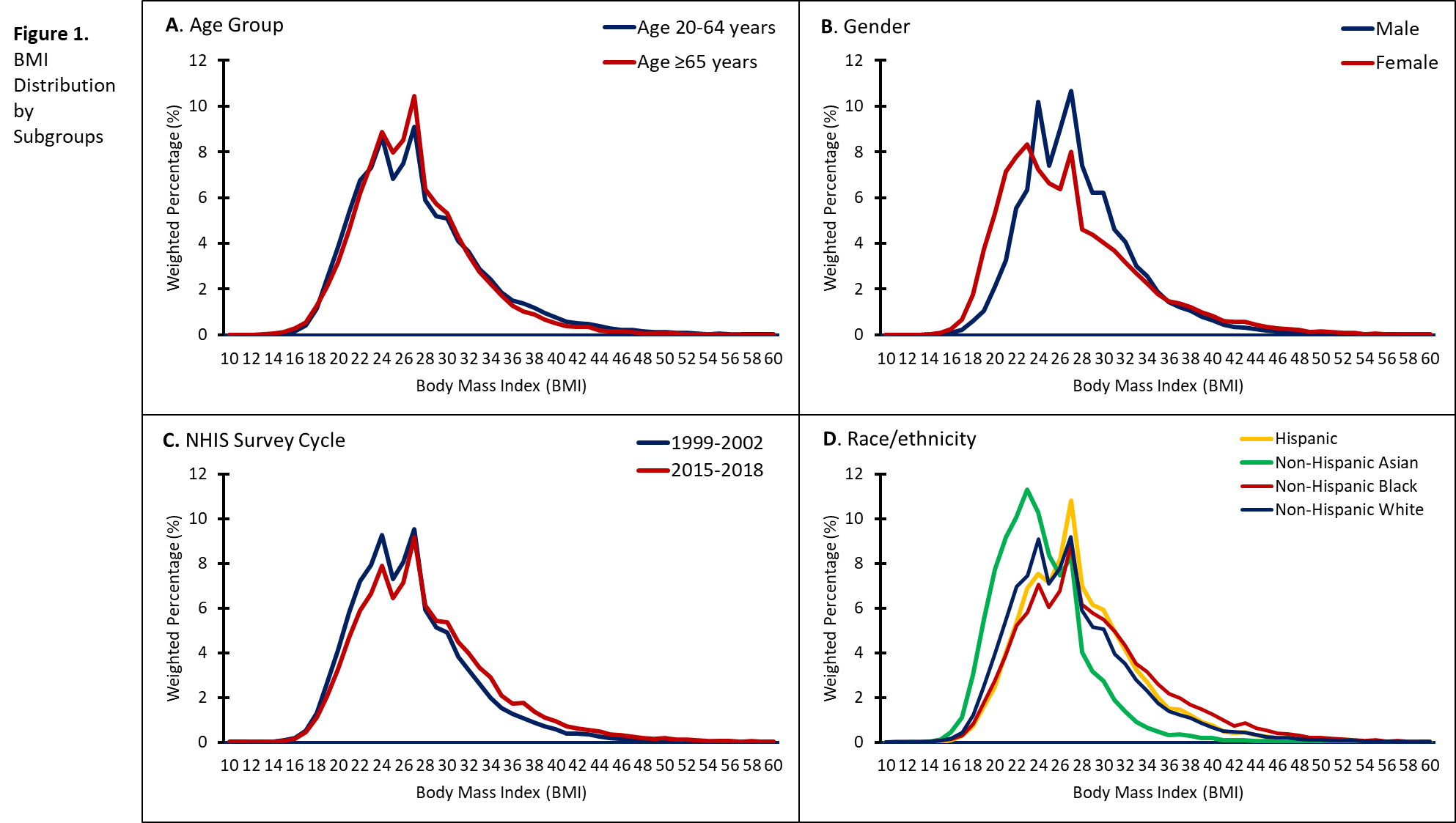


| Supplementary Table S1. Mortality Rates by Subgroups (NHIS 1999-2018) | | | | | | | | | | |
| --- | --- | --- | --- | --- | --- | --- | --- | --- | --- | --- |
|  | **<18.5 kg/m2** | **18.5-19.9** | **20.0-22.4** | **22.5-24.9** | **25.0-27.4** | **27.5-29.9** | **30.0-34.9** | **35.0-39.9** | **≥40** |  |
| Total N = 554,332 | 10,316 | 21,648 | 74,485 | 103,441 | 115,601 | 77,835 | 93,002 | 35,347 | 22,657 |  |
| Overall |  |  |  |  |  |  |  |  |  |  |
| Median follow-up time in years (IQR) | 7.5 (3.1-13.4) | 8.8 (4.2-14.0) | 8.8 (4.2-14.1) | 8.7 (4.2-13.9) | 8.6 (4.1-13.7) | 8.2 (3.9-13.3) | 7.9 (3.7-12.8) | 7.3 (3.4-12.2) | 7.0 (3.2-11.6) |  |
| Person-years of follow-up | 91442 | 212148 | 735583 | 1017182 | 1119744 | 737615 | 855177 | 311595 | 192354 |  |
| Number of Deaths in 5 years | 1543 | 1655 | 4657 | 5782 | 5977 | 3740 | 4441 | 1712 | 1264 |  |
| 5-year all-cause mortality incidence rate (per 1,000 person-years) | 35.2 (33.5-37.0) | 17.1 (16.3-18.0) | 13.9 (13.5-14.3) | 12.4 (12.1-12.7) | 11.5 (11.2-11.8) | 10.7 (10.4-11.1) | 10.7 (10.4-11.0) | 10.9 (10.4-11.0) | 12.7 (12.0-13.4) |  |
| Number of Total Deaths | 2518 | 3263 | 10091 | 14165 | 15694 | 10153 | 12200 | 4495 | 3228 |  |
| All-year all-cause mortality rate (per 1,000 person-years) | 27.5 (26.5-28.6) | 15.4 (14.9-15.9) | 13.7 (13.5-14.0) | 13.9 (13.7-14.2) | 14.0 (13.8-14.2) | 13.8 (13.5-14.0) | 14.3 (14.0-14.5) | 14.4 (14.0-14.9) | 16.8 (16.2-17.4) |  |
| Male |  |  |  |  |  |  |  |  |  |  |
| Median follow-up time in years (IQR) | 6.6 (2.6-12.4) | 7.9 (3.4-12.9) | 8.4 (3.8-13.7) | 8.7 (4.1-13.9) | 8.7 (4.1-13.8) | 8.4 (4.0-13.4) | 7.9 (3.7-12.8) | 7.2 (3.3-11.9) | 6.8 (3.1-11.3) |  |
| Person-years of follow-up | 18403 | 43799 | 236311 | 454325 | 613899 | 412781 | 410988 | 125659 | 58864 |  |
| Number of Deaths in 5 years | 449 | 528 | 2104 | 3038 | 3368 | 2138 | 2313 | 821 | 457 |  |
| 5-year all-cause mortality incidence rate (per 1,000 person-years) | 48.6 (44.3-53.3) | 25.1 (23.0-27.3) | 19.0 (18.2-19.8) | 14.5 (14.0-15.1) | 11.9 (11.5-12.3) | 11.0 (10.5-11.4) | 11.5 (11.1-12.0) | 12.8 (11.9-13.7) | 14.6 (13.3-16.0) |  |
| Number of Total Deaths | 651 | 906 | 4062 | 6869 | 8524 | 5620 | 6042 | 1956 | 1089 |  |
| All-year all-cause mortality rate (per 1,000 person-years) | 35.4 (32.8-38.2) | 20.7 (19.4-22.1) | 17.2 (16.7-17.7) | 15.1 (14.8-15.5) | 13.9 (13.6-14.2) | 13.6 (13.3-14.0) | 14.7 (14.3-15.1) | 15.6 (14.9-16.3) | 18.5 (17.4-19.6) |  |
| Female |  |  |  |  |  |  |  |  |  |  |
| Median follow-up time in years (IQR) | 7.8 (3.4-13.7) | 9.2 (4.5-14.3) | 9.0 (4.4-14.2) | 8.7 (4.2-13.9) | 8.4 (4.0-13.4) | 8.1 (3.8-13.1) | 7.8 (3.7-12.8) | 7.5 (3.4-12.4) | 7.1 (3.3-11.8) |  |
| Person-years of follow-up | 73039 | 168349 | 499272 | 562857 | 505845 | 324834 | 444189 | 185936 | 133490 |  |
| Number of Deaths in 5 years | 1094 | 1127 | 2553 | 2744 | 2609 | 1602 | 2128 | 891 | 807 |  |
| 5-year all-cause mortality incidence rate (per 1,000 person-years) | 31.7 (29.8-33.6) | 14.9 (14.1-15.8) | 11.4 (11.0-11.9) | 10.6 (10.2-11.0) | 11.0 (10.6-11.5) | 10.4 (9.9-10.9) | 9.9 (9.5-10.4) | 9.7 (9.1-10.3) | 11.9 (11.1-12.7) |  |
| Number of Total Deaths | 1867 | 2357 | 6029 | 7296 | 7170 | 4533 | 6158 | 2539 | 2139 |  |
| All-year all-cause mortality rate (per 1,000 person-years) | 25.6 (24.4-26.7) | 14.0 (13.4-14.6) | 12.1 (11.8-12.4) | 13.0 (12.7-13.3) | 14.2 (13.9-14.5) | 14.0 (13.6-14.4) | 13.9 (13.5-14.2) | 13.7 (13.1-14.2) | 16.0 (15.4-16.7) |  |
| Age 20-64 years |  |  |  |  |  |  |  |  |  |  |
| Median follow-up time in years (IQR) | 9.0 (4.1-14.4) | 9.7 (4.9-14.7) | 9.6 (4.8-14.7) | 9.5 (4.7-14.6) | 9.3 (4.6-14.3) | 8.9 (4.3-13.9) | 8.5 (4.1-13.4) | 7.8 (3.6-12.6) | 7.3 (3.4-12.0) |  |
| Person-years of follow-up | 76649 | 184953 | 634034 | 847422 | 923204 | 608481 | 713330 | 268161 | 171988 |  |
| Number of Deaths | 311 | 328 | 941 | 1260 | 1573 | 1126 | 1512 | 712 | 665 |  |
| 5-year all-cause mortality incidence rate (per 1,000 person-years) | 11.3  (9.9-12.6) | 4.8  (4.3-5.4) | 4.8  (4.5-5.1) | 4.8  (4.5-5.1) | 5.3  (5.1-5.6) | 5.7  (5.4-6.0) | 6.1  (5.8-6.4) | 7.3  (6.7-7.9) | 9.6  (8.9-10.5) |  |
| Number of Total Deaths | 652 | 879 | 2804 | 3928 | 5053 | 3541 | 5030 | 2216 | 1971 |  |
| All-year all-cause mortality rate (per 1,000 person-years) | 8.5 (7.9-9.2) | 4.8 (4.4-5.1) | 4.4 (4.3-4.6) | 4.6 (4.5-4.8) | 5.5 (5.3-5.6) | 5.8 (5.6-6.0) | 7.1 (6.9-7.3) | 8.3 (7.9-8.6) | 11.5 (11.0-12.0) |  |
| Age 65+ years |  |  |  |  |  |  |  |  |  |  |
| Median follow-up time in years (IQR) | 3.3 (1.1-6.9) | 4.8 (1.9-8.8) | 5.2 (2.2-9.6) | 5.7 (2.6-10.0) | 5.9 (2.7-10.2) | 5.8 (2.6-10.1) | 5.5 (2.5-9.8) | 5.1 (2.3-9.2) | 4.7 (2.0-8.7) |  |
| Person-years of follow-up | 14793 | 27195 | 101549 | 169760 | 196540 | 129134 | 141847 | 43434 | 20366 |  |
| Number of Deaths | 1232 | 1327 | 3716 | 4522 | 4404 | 2614 | 2929 | 1000 | 599 |  |
| 5-year all-cause mortality incidence rate (per 1,000 person-years) | 124.5 (117.7-131.6) | 79.4 (75.2-83.8) | 62.1 (60.1-64.1) | 46.6  (45.2-48.0) | 39.3 (38.1-40.5) | 35.1 (33.8-36.5) | 35.1 (33.8-36.4) | 37.6 (35.4-40.0) | 46.0 (42.4-49.8) |  |
| Number of Total Deaths | 1338 | 1635 | 4893 | 6636 | 6723 | 4082 | 4259 | 1351 | 728 |  |
| All-year all-cause mortality rate (per 1,000 person-years) | 126.1 (120.5-132.0) | 87.7 (84.2-91.3) | 71.8 (70.1-73.4) | 60.3 (59.1-61.5) | 54.1 (53.1-55.2) | 51.2 (50.0-52.5) | 50.5 (49.4-51.7) | 52.5 (50.4-54.7) | 61.7 (58.4-65.2) |  |

In both female and male adults, 5-year mortality was highest in those with BMI <18.5 (females <65: 7.2 [95% CI, 6.1-8.5] per 1,000 person-years, females ≥65: 112 [104-121], males <65: 18.3 [15-22], males ≥65: 183 [162-208]). Five-year mortality was lowest at 2.9 among BMI of 20.0-22.4 in females <65, 5.1 at BMI of 25.0-27.4 in males <65, 31 at BMI of 30.0-34.9 in females ≥65, and 39 at BMI of 27.5-29.9 in males ≥65 (**Supplementary** **Table S1**).

Among non-Hispanic White, non-Hispanic Black, and Hispanic participants, 5-year mortality was highest in those with BMI <18.5 (White: 40 [95% CI, 38-43] per 1,000 person-years, Black: 43 [37-50], Hispanic: 20 [16-25]). Five-year mortality was lowest at 11.7 (11.2-12.2) among those with BMI of 30.0-34.9 in White adults and 10.0 (8.8-11.3) at BMI of 35.0-39.9 in Black adults, and 7.1 [6.5-7.8] in Hispanic adults.

| Supplementary Table S2. Mortality Rates by Survey Cycle Year (NHIS 1999-2018) | | | | | | | | | | |
| --- | --- | --- | --- | --- | --- | --- | --- | --- | --- | --- |
|  | **<18.5 kg/m2** | **18.5-19.9** | **20.0-22.4** | **22.5-24.9** | **25.0-27.4** | **27.5-29.9** | **30.0-34.9** | **35.0-39.9** | **≥40** |  |
| Total N = 554,332 | 10,316 | 21,648 | 74,485 | 103,441 | 115,601 | 77,835 | 93,002 | 35,347 | 22,657 |  |
| 1999-2002 |  |  |  |  |  |  |  |  |  |  |
| 3-year all-cause mortality incidence rate (per 1,000 person-years) | 37.7 (33.3-42.6) | 17.0 (15.0-19.2) | 12.7 (11.7-13.7) | 11.3 (10.6-12.2) | 10.1 (9.4-10.8) | 10.0 (9.1-11.0) | 9.3 (8.5-10.1) | 9.5 (8.1-11.0) | 11.9 (9.9-14.3) |  |
| All-year all-cause mortality rate (per 1,000 person-years) | 22.6 (21.1-24.2) | 14.2 (13.4-15.0) | 13.2 (12.8-13.7) | 13.9 (13.6-14.3) | 14.6 (14.2-14.9) | 14.8 (14.3-15.2) | 15.6 (15.1-16.1) | 15.9 (15.1-16.8) | 18.7 (17.6-19.9) |  |
| 2003-2006 |  |  |  |  |  |  |  |  |  |  |
| 3-year all-cause mortality incidence rate (per 1,000 person-years) | 38.1 (33.3-43.7) | 19.0 (16.7-21.6) | 14.0 (12.9-15.2) | 11.8 (11.0-12.7) | 10.4 (9.7-11.2) | 9.0 (8.1-9.9) | 9.2 (8.4-10.1) | 9.9 (8.5-11.5) | 9.3 (7.7-11.3) |  |
| All-year all-cause mortality rate (per 1,000 person-years) | 24.7 (22.7-26.8) | 14.4 (13.5-15.5) | 13.0 (12.5-13.5) | 13.8 (13.4-14.3) | 14.0 (13.5-14.4) | 14.2 (13.6-14.7) | 14.5 (14.0-15.0) | 14.6 (13.8-15.5) | 17.1 (16.0-18.3) |  |
| 2007-2010 |  |  |  |  |  |  |  |  |  |  |
| 3-year all-cause mortality incidence rate (per 1,000 person-years) | 36.8 (31.8-42.7) | 14.9 (12.7-17.4) | 13.1 (12.0-14.4) | 11.8 (10.9-12.8) | 10.2 (9.4-11.1) | 9.6 (8.6-10.6) | 8.9 (8.1-9.8) | 11.0 (9.6-12.7) | 10.8 (9.0-12.8) |  |
| All-year all-cause mortality rate (per 1,000 person-years) | 30.2 (27.5-33.1) | 16.1 (14.8-17.5) | 13.5 (12.8-14.2) | 13.7 (13.2-14.3) | 13.1 (12.6-13.6) | 12.5 (11.9-13.1) | 13.1 (12.6-13.7) | 13.2 (12.3-14.1) | 15.9 (14.7-17.2) |  |
| 2011-2014 |  |  |  |  |  |  |  |  |  |  |
| 3-year all-cause mortality incidence rate (per 1,000 person-years) | 38.9 (34.4-43.9) | 16.2 (14.2-18.4) | 14.3 (13.2-15.4) | 11.6 (10.9-12.5) | 11.2 (10.5-11.9) | 10.0 (9.2-10.9) | 10.1 (9.4-10.9) | 10.2 (9.0-11.4) | 13.0 (11.4-14.8) |  |
| All-year all-cause mortality rate (per 1,000 person-years) | 35.1 (13.1-38.5) | 16.8 (15.4-18.3) | 14.9 (14.1-15.6) | 13.9 (13.3-14.5) | 13.8 (13.2-14.4) | 12.5 (11.8-13.1) | 12.9 (12.3-13.5) | 13.5 (12.6-14.5) | 15.5 (14.2-16.8) |  |
| 2015-2018 |  |  |  |  |  |  |  |  |  |  |
| 3-year all-cause mortality incidence rate (per 1,000 person-years) | 45.5 (39.8-52.0) | 22.2 (19.4-25.4) | 16.1 (14.9-17.6) | 12.7 (11.7-13.7) | 12.0 (11.2-13.0) | 11.2 (10.2-12.3) | 11.6 (10.7-12.6) | 11.0 (9.6-12.5) | 12.4 (10.7-14.4) |  |
| All-year all-cause mortality rate (per 1,000 person-years) | 47.6 (41.9-54.1) | 23.9 (21.1-27.2) | 18.8 (17.4-20.3) | 14.9 (13.9-16.0) | 14.1 (13.2-15.1) | 13.3 (12.2-14.5) | 13.4 (12.5-14.5) | 13.0 (11.5-14.6) | 14.3 (12.5-16.4) |  |


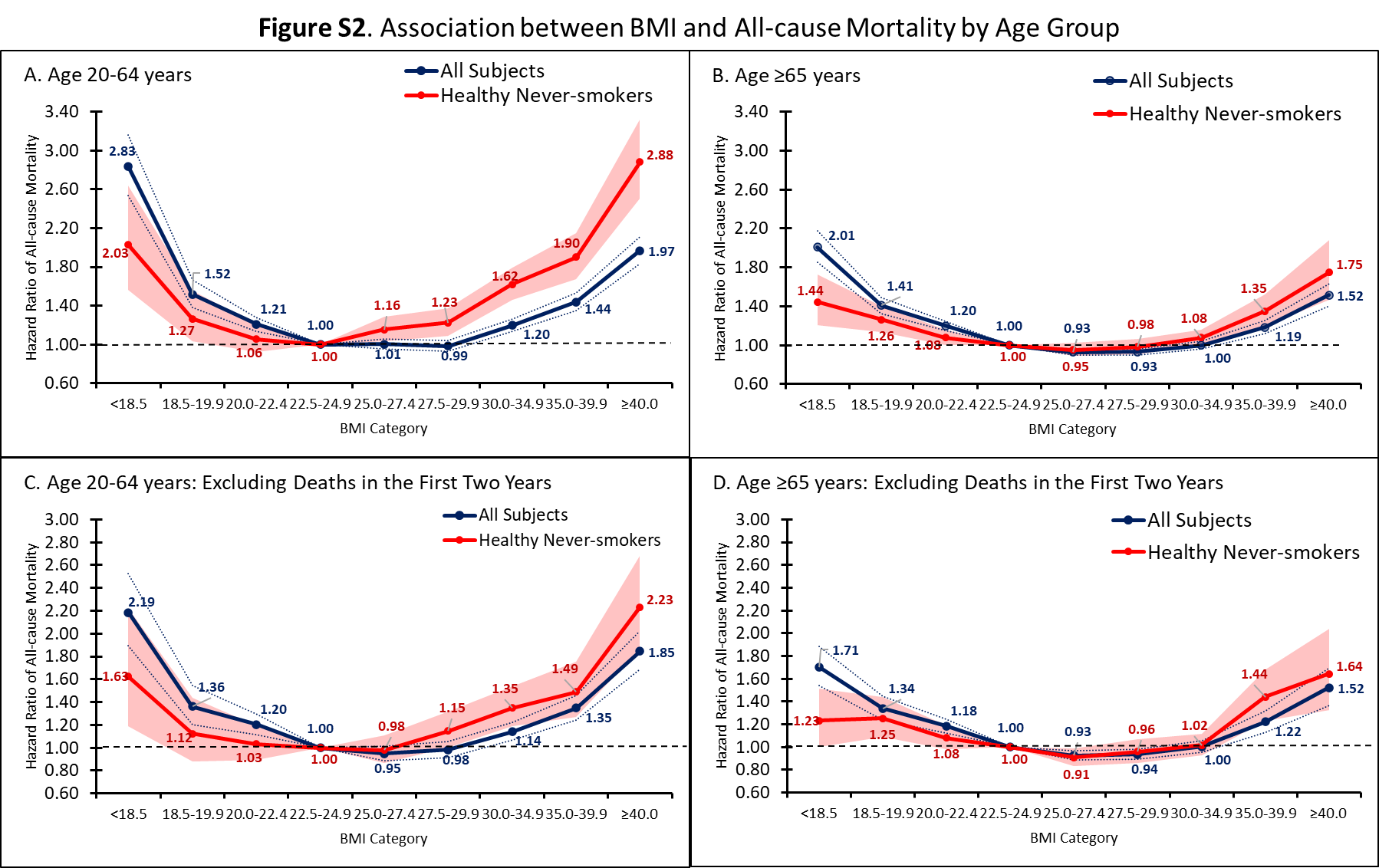


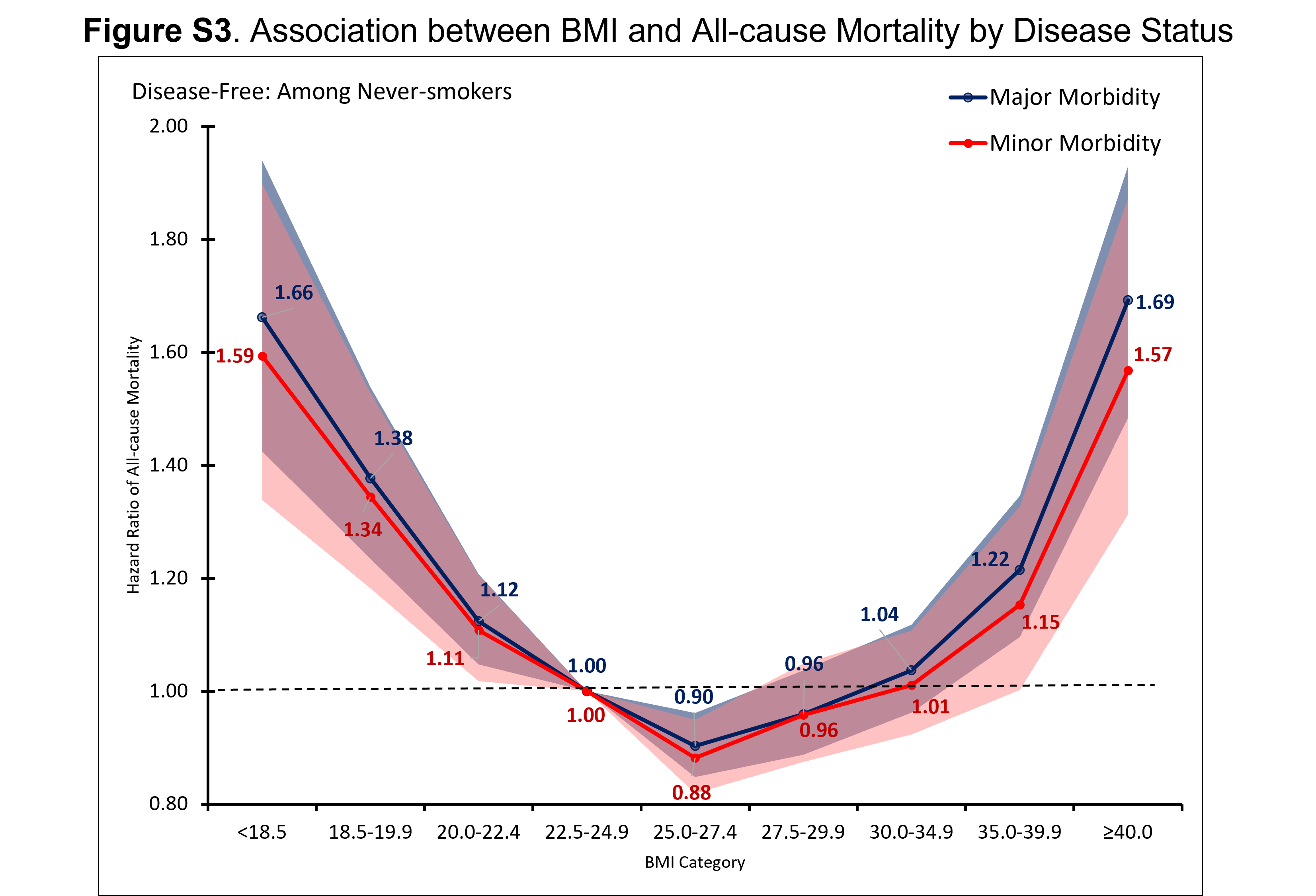


| Supplementary Table S3. 1999-2018 NHANES Baseline Characteristics by BMI Category | | | | | | | | |
| --- | --- | --- | --- | --- | --- | --- | --- | --- |
| NHANES 1999-2018 |  | **Body Mass Index (BMI) Category** | | | | | | |
| Variables | **Overall** | **<18.5 kg/m2** | **18.5-22.5** | **22.5-24.9** | **25-27.4** | **27.5-29.9** | **30-34.9** | **>=35** |
| TOTAL N = 44,308 | 44,308 | 578 | 6,369 | 6,841 | 7,852 | 7,248 | 9,162 | 6,836 |
| Demographics/Sociobehavioral Factors |  |  |  |  |  |  |  |  |
| Age, Mean (SD) | 46.7 (0.2) | 41.3 (0.8) | 42.8 (0.4) | 45.7 (0.3) | 47.8 (0.3) | 48.7 (0.3) | 48.5 (0.3) | 47.1 (0.3) |
| >=65 (%) | 8506 (17%) | 131 (16%) | 1188 (14%) | 1173 (17%) | 1702 (18%) | 1566 (19%) | 1751 (18%) | 995 (15%) |
| Sex (% Female) | 17286 (51%) | 348 (67%) | 3095 (61%) | 2113 (50%) | 2709 (44%) | 2378 (41%) | 3483 (47%) | 3160 (62%) |
| Race/Ethnicity |  |  |  |  |  |  |  |  |
| Mexican American | 6153 (7.9%) | 35 (2.6%) | 647 (5.2%) | 708 (6.6%) | 1251 (8.7%) | 1200 (9.2%) | 1449 (9.5%) | 863 (8.3%) |
| Other Hispanic | 2629 (5.3%) | 20 (2.8%) | 342 (4.3%) | 321 (4.9%) | 531 (6.2%) | 489 (6.1%) | 579 (5.6%) | 347 (4.7%) |
| non-Hispanic White | 16402 (70%) | 302 (72%) | 2840 (72%) | 2281 (72%) | 2964 (70%) | 2700 (71%) | 3165 (69%) | 2150 (66%) |
| non-Hispanic Black | 7117 (11%) | 134 (11%) | 972 (8.8%) | 749 (8.2%) | 1115 (9.1%) | 1046 (9.7%) | 1592 (12%) | 1509 (17%) |
| Other/Multiracial | 2505 (5.9%) | 87 (11%) | 751 (9.9%) | 485 (8.0%) | 446 (5.6%) | 279 (4.3%) | 293 (3.8%) | 164 (3.5%) |
| Education |  |  |  |  |  |  |  |  |
| <High School | 9726 (18%) | 167 (23%) | 1302 (16%) | 1169 (16%) | 1851 (19%) | 1698 (19%) | 2124 (20%) | 1415 (19%) |
| High School | 8084 (24%) | 131 (22%) | 1242 (22%) | 988 (22%) | 1388 (22%) | 1343 (25%) | 1729 (25%) | 1263 (27%) |
| >High School | 16995 (58%) | 280 (55%) | 3008 (62%) | 2387 (62%) | 3068 (59%) | 2672 (55%) | 3225 (55%) | 2355 (54%) |
| Lowest Tertile Poverty-Income Ratio | 12557 (26%) | 289 (42%) | 2027 (27%) | 1526 (25%) | 2189 (24%) | 1995 (25%) | 2571 (26%) | 1960 (30%) |
| Smoking Status (Smoked>100 cigarettes?) | 16341 (47%) | 330 (54%) | 2618 (48%) | 2122 (46%) | 3000 (48%) | 2751 (48%) | 3298 (48%) | 2222 (45%) |
| Marital Status |  |  |  |  |  |  |  |  |
| Never Married | 6097 (18%) | 188 (33%) | 1432 (26%) | 824 (19%) | 961 (15%) | 730 (13%) | 1036 (14%) | 926 (18%) |
| Separated/Divorced/Widowed | 7625 (18%) | 133 (20%) | 1132 (17%) | 935 (18%) | 1346 (18%) | 1241 (18%) | 1613 (19%) | 1225 (21%) |
| Married | 18301 (57%) | 206 (38%) | 2523 (50%) | 2410 (55%) | 3469 (59%) | 3258 (62%) | 3921 (61%) | 2514 (55%) |
| Other | 2389 (7.1%) | 44 (8.2%) | 413 (7.8%) | 319 (7.8%) | 453 (7.5%) | 412 (7.2%) | 427 (5.8%) | 321 (6.6%) |
| Total caloric intake (kcal/day) | 2142 (7) | 2179 (61) | 2149 (17) | 2152 (17) | 2145 (16) | 2197 (16) | 2123 (15) | 2079 (14) |
| Total Carbohydrate Intake (g/day) | 260 (0.9) | 279 (9) | 268 (2) | 264 (2) | 262 (2) | 262 (2) | 254 (2) | 250 (2) |
| Total Protein Intake (g/day) | 82.7 (0.3) | 79 (2) | 79.6 (0.7) | 82.6 (0.8) | 83.2 (0.7) | 85.8 (0.7) | 83.6 (0.6) | 82 (0.7) |
| Total Fat Intake (g/day) | 81.1 (0.4) | 78 (3) | 78.8 (0.8) | 79.5 (0.8) | 79.7 (0.7) | 84.1 (0.8) | 82.2 (0.7) | 82.1 (0.8) |
| Total Fiber (g/day) | 16.9 (0.1) | 16.2 (0.7) | 17.5 (0.3) | 17.5 (0.2) | 17.5 (0.2) | 16.9 (0.2) | 16.3 (0.2) | 15.7 (0.1) |
| Sodium intake < 1.5 g/day (%)  Missing = 4,452 | 2343 (5.2%) | 43 (6.5%) | 347 (4.9%) | 289 (5.2%) | 421 (5.5%) | 387 (4.9%) | 503 (5.3%) | 353 (5.3%) |
| Excess Alcohol consumption (%) | 11068 (35%) | 210 (40%) | 1941 (39%) | 1460 (36%) | 1978 (34%) | 1804 (35%) | 2154 (33%) | 1521 (32%) |
| Physical Activity (>600 MET-min/week) Missing = 21,682 | 9047 (71%) | 127 (68%) | 1540 (73%) | 1274 (73%) | 1682 (72%) | 1474 (72%) | 1779 (70%) | 1171 (63%) |
| Any vigorous physical activity (%)  Missing = 6,848 | 8579 (35%) | 133 (33%) | 1723 (43%) | 1394 (43%) | 1727 (38%) | 1361 (34%) | 1451 (30%) | 790 (22%) |
| Maximum BMI | 30.5 (0.1) | 21.0 (0.2) | 23.6 (0.1) | 26.2 (0.1) | 28.3 (0.1) | 30.8 (0.1) | 34.2 (0.1) | 42.2 (0.1) |
| Weight change in past year  Missing = 862 |  |  |  |  |  |  |  |  |
| >= 10 pounds | 6512 (19%) | 29 (5.2%) | 547 (9.7%) | 596 (13%) | 1012 (17%) | 1076 (20%) | 1743 (25%) | 1509 (30%) |
| -10 to 10 pounds | 20995 (63%) | 453 (82%) | 4201 (78%) | 3228 (72%) | 4046 (65%) | 3362 (60%) | 3625 (53%) | 2080 (45%) |
| <-10 pounds (unintentional) | 2424 (5.9%) | 78 (11%) | 528 (7.9%) | 337 (6.6%) | 434 (5.9%) | 371 (5.1%) | 420 (5.1%) | 256 (4.3%) |
| <-10 pounds (intentional) | 4013 (12%) | 8 (2.0%) | 178 (4.0%) | 308 (8.6%) | 675 (12%) | 761 (15%) | 1105 (16%) | 978 (20%) |
| General Health Condition |  |  |  |  |  |  |  |  |
| Very Good/Excellent | 14503 (50%) | 260 (53%) | 2899 (61%) | 2277 (59%) | 2971 (56%) | 2380 (50%) | 2426 (41%) | 1290 (29%) |
| Fair/Good | 18719 (47%) | 274 (42%) | 2346 (36.6%) | 2088 (38%) | 3123 (41%) | 3114 (47%) | 4289 (55%) | 3374 (65%) |
| Poor | 1565 (3.2%) | 44 (5.9%) | 194 (2.4%) | 177 (2.5%) | 208 (2.4%) | 217 (2.4%) | 359 (3.5%) | 366 (5.7%) |
| Health Service Factors |  |  |  |  |  |  |  |  |
| Insurance Coverage (%) | 26,876 (81%) | 415 (72%) | 4187 (79%) | 3502 (81%) | 4845 (81%) | 4414 (81%) | 5549 (82%) | 3964 (82%) |
| Number of Healthcare Visits in Past Year |  |  |  |  |  |  |  |  |
| 0 | 5839 (16%) | 113 (19%) | 1006 (17%) | 846 (18%) | 1180 (18%) | 983 (17%) | 1074 (15%) | 637 (12%) |
| 1-3 | 15592 (47%) | 252 (45%) | 2649 (50%) | 2122 (49%) | 2916 (49%) | 2658 (48%) | 2997 (44%) | 1998 (40%) |
| 4+ | 13353 (37%) | 212 (36%) | 1893 (33%) | 1570 (33%) | 2210 (33%) | 2071 (35%) | 3003 (41%) | 2394 (47%) |
| Mental Health Visit in Past 12 Months | 2498 (7.9%) | 57 (12%) | 440 (9.1%) | 301 (7.4%) | 399 (7.6%) | 339 (6.5%) | 508 (7.2%) | 454 (9.2%) |
| Examination Measurements |  |  |  |  |  |  |  |  |
| Heart Rate (beats-per-min) | 72.3 (0.1) | 73.8 (0.8) | 72.1 (0.2) | 70.6 (0.3) | 70.9 (0.2) | 71.4 (0.2) | 72.9 (0.2) | 76.1 (0.2) |
| Standing Height (m) | 168.9 (0.1) | 166.9 (0.4) | 168.1 (0.2) | 169.2 (0.2) | 169.7 (0.2) | 170.0 (0.2) | 169.3 (0.2) | 167.5 (0.2) |
| Mean Weight (kg) | 81.4 (0.2) | 48.9 (0.3) | 60.2 (0.1) | 68.9 (0.2) | 75.8 (0.2) | 83.3 (0.2) | 92.6 (0.2) | 113.7 (0.4) |
| Waist Circumference (cm) | 97.5 (0.2) | 70.6 (0.3) | 79.4 (0.1) | 87.2 (0.1) | 93.4 (0.1) | 99.9 (0.1) | 107.5 (0.1) | 122.6 (0.2) |
| Arm circumference (cm) | 33.0 (0.04) | 23.7 (0.1) | 27.5 (0.1) | 30.1 (0.1) | 31.9 (0.1) | 33.8 (0.1) | 35.9 (0.1) | 40.6 (0.1) |
| Relative Fat Mass Index | 34.5 (0.1) | 24.5 (0.4) | 28.8 (0.1) | 31.1 (0.1) | 32.8 (0.1) | 34.7 (0.1) | 37.9 (0.1) | 43.9 (0.1) |
| Mean Systolic Blood Pressure (mmHg) | 122 (0.2) | 115 (1) | 117 (0.3) | 120 (0.3) | 122 (0.3) | 123 (0.3) | 125 (0.3) | 126 (0.3) |
| Mean Diastolic Blood Pressure (mmHg) | 71 (0.2) | 68 (0.5) | 69 (0.2) | 70 (0.2) | 71 (0.2) | 72 (0.2) | 73 (0.2) | 72 (0.3) |
| Laboratory Measurements |  |  |  |  |  |  |  |  |
| Hemoglobin A1c (%) | 5.54 (0.01) | 5.23 (0.01) | 5.29 (0.01) | 5.37 (0.01) | 5.46 (0.01) | 5.55 (0.01) | 5.7 (0.02) | 5.94 (0.02) |
| Serum HDL (mg/dl) | 52.5 (0.2) | 64.2 (0.9) | 61.6 (0.4) | 56.7 (0.4) | 52.9 (0.3) | 49.7 (0.3) | 47.3 (0.2) | 46.1 (0.2) |
| Serum triglycerides (mg/dl) | 149 (1) | 88 (3) | 103 (1) | 122 (2) | 147.2 (1.9) | 165 (2) | 183 (2) | 176.4 (2.3) |
| Serum ALT (mg/dl) | 25.8 (0.2) | 20.0 (1) | 21.1 (0.5) | 23.2 (0.3) | 25.6 (0.3) | 27.2 (0.3) | 28.7 (0.3) | 29.2 (0.5) |
| Albumin (g/dl) | 4.31 (0.01) | 4.44 (0.01) | 4.40 (0.01) | 4.37 (0.01) | 4.36 (0.01) | 4.32 (0.01) | 4.26 (0.01) | 4.10 (0.01) |
| Hemoglobin (g/dl) | 14.4 (0.02) | 14.1 (0.1) | 14.1 (0.1) | 14.4 (0.1) | 14.5 (0.1) | 14.57 (0.1) | 14.5 (0.1) | 14.1 (0.1) |
| Total Bilirubin (mg/dl) | 0.72 (0.01) | 0.76 (0.02) | 0.75 (0.01) | 0.74 (0.01) | 0.75 (0.01) | 0.72 (0.01) | 0.71 (0.01) | 0.64 (0.01) |
| Prognostic Nutrition Index (PNI) | 43.1 (0.04) | 44.4 (0.2) | 44.0 (0.1) | 43.7 (0.1) | 43.57 (0.06) | 43.22 (0.06) | 42.6 (0.1) | 41.1 (0.1) |
| Serum Vitamin D | 67.7 (0.5) | 70.8 (1.9) | 73.4 (0.7) | 72.6 (0.7) | 70.2 (0.6) | 67.7 (0.6) | 64.0 (0.7) | 57.9 (0.6) |
| RBC Folate | 424 (4) | 373 (12) | 394 (5) | 408 (5) | 423 (5) | 431 (6) | 439 (6) | 459 (6) |
| Comorbidities |  |  |  |  |  |  |  |  |
| MACE (%) | 3443 (7.6%) | 46 (6.2%) | 381 (4.7%) | 391 (5.8%) | 605 (7.3%) | 585 (8.1%) | 818 (9.3%) | 617 (10%) |
| History of Stroke | 1218 (2.6%) | 17 (2.7%) | 143 (1.8%) | 152 (2.2%) | 208 (2.3%) | 200 (2.7%) | 293 (3.1%) | 205 (3.3%) |
| History of Myocardial Infarction | 1476 (3.3%) | 23 (2.8%) | 156 (1.9%) | 168 (2.6%) | 255 (3.2%) | 248 (3.3%) | 378 (4.5%) | 248 (4.3%) |
| History of Heart Failure | 1063 (2.2%) | 12 (1.4%) | 102 (1.3%) | 110 (1.5%) | 158 (1.8%) | 173 (2.3%) | 250 (2.5%) | 258 (4.4%) |
| History of Coronary Heart Disease | 1426 (3.3%) | 15 (2.1%) | 142 (1.8%) | 172 (2.7%) | 262 (3.2%) | 259 (3.9%) | 351 (4.3%) | 225 (4.1%) |
| Metabolic Syndrome (%) | 14597 (38%) | 28 (3.7%) | 534 (7.4%) | 969 (17%) | 2161 (30%) | 2800 (44%) | 4476 (60%) | 3629 (71%) |
| Impaired glucose tolerance (%) | 14694 (35%) | 127 (17%) | 1295 (18%) | 1454 (25%) | 2443 (32%) | 2636 (39%) | 3653 (44%) | 3086 (57%) |
| Diabetes (%) | 5163 (11%) | 19 (2.0%) | 304 (3.5%) | 396 (5.3%) | 737 (7.4%) | 856 (10%) | 1401 (15%) | 1450 (25%) |
| Hypertriglyceridemia (%)  Missing = 1,952 | 11603 (35%) | 51 (9.5%) | 830 (15%) | 1090 (23%) | 2064 (33%) | 2320 (42%) | 3108 (47%) | 2140 (49%) |
| Low HDL cholesterol (%)  Missing = 4,274 | 14415 (45%) | 100 (20%) | 1192 (23%) | 1436 (33%) | 2509 (43%) | 2573 (50%) | 3685 (58%) | 2920 (66%) |
| Elevated Waist Circum. (%)  Missing = 1,012 | 18281 (51%) | 1 (0.3%) | 172 (3.0%) | 121 (2.0%) | 2375 (37%) | 3789 (67%) | 6457 (92%) | 4805 (100%) |
| Hypertension (%) | 14409 (36%) | 148 (21%) | 1461 (21%) | 1433 (25%) | 2429 (33%) | 2511 (39%) | 3537 (45%) | 2890 (54%) |
| Chronic Kidney Disease (%)  Missing = 1,930 | 3209 (7.9%) | 33 (4.5%) | 389 (5.9%) | 382 (6.7%) | 618 (8.5%) | 575 (8.8%) | 714 (8.4%) | 498 (9.5%) |
| Asthma (%) | 2391 (6.9%) | 32 (4.8%) | 285 (5.0%) | 250 (6.4%) | 342 (5.8%) | 328 (5.9%) | 547 (7.9%) | 607 (11%) |
| Emphysema (%) | 697 (1.8%) | 36 (6.1%) | 126 (1.7%) | 86 (1.5%) | 124 (1.7%) | 104 (1.6%) | 122 (1.5%) | 99 (2.2%) |
| Chronic Bronchitis (%) | 2015 (6.0%) | 54 (9.2%) | 264 (4.9%) | 198 (4.6%) | 296 (4.8%) | 296 (5.6%) | 406 (6.2%) | 501 (10%) |
| Liver condition (%) | 588 (1.5%) | 13 (1.5%) | 80 (1.2%) | 63 (1.1%) | 90 (1.2%) | 88 (1.3%) | 131 (1.7%) | 123 (2.7%) |
| Non-Skin Cancer/Malignancy (%) | 3209 (9.1%) | 53 (9.7%) | 451 (8.1%) | 458 (10%) | 622 (9.2%) | 517 (8.9%) | 674 (9.6%) | 434 (9.0%) |
| Functional Limitations (%) | 4551 (10%) | 111 (15%) | 585 (7.9%) | 477 (8.5%) | 695 (8.7%) | 678 (9.1%) | 1021 (11%) | 984 (16%) |
| Malnutrition (%)  Missing = 1,930 | 316 (0.7%) | 10 (1.1%) | 45 (0.6%) | 31 (0.5%) | 43 (0.5%) | 28 (0.3%) | 65 (0.6%) | 94 (1.8%) |
| Malnourishment (via PNI, %)  Missing = 2,048 | 15264 (42%) | 173 (26%) | 1774 (31%) | 1589 (34%) | 2395 (36%) | 2421 (40%) | 3576 (48%) | 3336 (67%) |
| Folate Deficiency (%)  Missing = 1,621 | 666 (1.7%) | 25 (4.2%) | 151 (2.4%) | 102 (2.3%) | 117 (1.7%) | 87 (1.2%) | 106 (1.3%) | 78 (1.2%) |
| Vitamin D Insufficiency (%)  Missing = 5,627 | 2029 (4.7%) | 53 (7.4%) | 264 (4.0%) | 183 (2.9%) | 275 (3.5%) | 258 (3.5%) | 436 (5.1%) | 560 (9.1%) |
| Anemia (%)  Missing = 1,446 | 2806 (5.9%) | 52 (5.3%) | 460 (6.3%) | 374 (6.2%) | 423 (4.8%) | 401 (4.9%) | 567 (5.5%) | 529 (8.2%) |
| Moderate to Severe Anemia (%) | 271 (0.6%) | 10 (1.1%) | 53 (0.8%) | 35 (0.5%) | 38 (0.3%) | 34 (0.5%) | 63 (0.7%) | 38 (0.5%) |
| Thyroid condition (%) | 1906 (5.8%) | 24 (5.3%) | 248 (4.5%) | 217 (4.8%) | 263 (4.5%) | 326 (5.6%) | 436 (6.7%) | 392 (8.9%) |
| Medication Use |  |  |  |  |  |  |  |  |
| Number of medications, mean (SD) | 1.85 (0.02) | 1.34 (0.09) | 1.24 (0.04) | 1.49 (0.04) | 1.67 (0.04) | 1.81 (0.05) | 2.18 (0.04) | 2.9 (0.1) |
| Chronic medication Use (%) | 18699 (45%) | 200 (35%) | 1997 (35%) | 141 (39%) | 2792 (43%) | 2703 (45%) | 3665 (50%) | 2909 (58%) |
| Cardiovascular Agent (%) | 10756 (26%) | 93 (12%) | 990 (13%) | 1058 (18%) | 1809 (24%) | 1878 (28%) | 2672 (33%) | 2256 (42%) |
| Antimetabolic Agents (%) | 7953 (20%) | 54 (8.1%) | 669 (9.7%) | 842 (15%) | 1399 (19%) | 1417 (21%) | 2012 (25%) | 1560 (29%) |
| Hormonal agents (%) | 4924 (16%) | 76 (16%) | 856 (18%) | 671 (17%) | 778 (14%) | 759 (14%) | 993 (15%) | 791 (18%) |
| Antineoplastic Agent (%) | 698 (2.2%) | 12 (1.9%) | 138 (2.6%) | 99 (2.5%) | 110 (1.9%) | 103 (1.8%) | 136 (2.0%) | 100 (2.2%) |
| Psychiatric medications (%) | 3519 (12%) | 59 (12%) | 437 (9.0%) | 393 (10%) | 544 (11%) | 543 (11%) | 800 (14%) | 743 (17%) |
| Anticoagulation (%) | 1746 (3.7%) | 28 (3.6%) | 184 (2.3%) | 206 (3.2%) | 290 (3.3%) | 289 (3.6%) | 426 (4.5%) | 323 (5.2%) |
| Polypharmacy (%) | 5454 (13%) | 56 (8.3%) | 488 (7.0%) | 524 (9.5%) | 829 (11%) | 895 (13%) | 1360 (16%) | 1302 (24%) |
| No Minor Morbidity (%) | 4509 (15%) | 124 (25%) | 1333 (27%) | 861 (21%) | 891 (16%) | 560 (11%) | 505 (8.5%) | 235 (4.8%) |
| No Medium Morbidity (%) | 10283 (32%) | 184 (36%) | 2104 (41%) | 1659 (38%) | 2052 (35%) | 1656 (30%) | 1713 (26%) | 915 (18%) |
| *Medium Morbidity definition: Presence of any one of the following: self-reported COPD, emphysema, chronic bronchitis, non-skin cancer, current thyroid condition, current liver disease, cardiovascular disease (CAD, HF, stroke, MI), diabetes, hypertension, microalbuminuria, chronic kidney disease, other kidney disease, asthma, chronic prescription medication use (use of chronic antivirals for hepatitis or HIV, cardiovascular agents, chronic GI agents, chronic CNS agents, biologicals, immunologic agents, chronic respiratory agents, antimetabolic agents, non-contraceptive or BPH-related hormones or hormone modifiers, anticoagulation, cancer agents), moderate to severe anemia (<10 g/dl), unintentional weight loss of >10 pounds in the past year. **Minor morbidity is medium morbidity conditions + dyslipidemia, functional limitations, mild anemia, impaired glucose tolerance, self-rated poor health. | | | | | | | | |

| Supplementary Table S4. Comparison of NHANES and NHIS Cohorts | | |
| --- | --- | --- |
|  | NHANES Cohort | NHIS Cohort |
| N | 44,308 | 554,332 |
| Age, mean (SE) | 47.1 (0.2) | 46.3 (0.1) |
| Sex (% Female) | 22,111 (51%) | 301,790 (50%) |
| Race/Ethnicity |  |  |
| Non-Hispanic White | 19,645 (69%) | 354,870 (69%) |
| Non-Hispanic Black | 9,187 (11%) | 77,647 (12%) |
| BMI, mean (SE) | 28.7 (0.06) | 27.5 (0.01) |
| BMI by 4-year survey cycle |  |  |
| 1999-2002 | 27.9 (0.1) | 26.7 (0.02) |
| 2003-2006 | 28.2 (0.1) | 27.1 (0.03) |
| 2007-2010 | 28.6 (0.1) | 27.6 (0.03) |
| 2011-2014 | 28.8 (0.1) | 27.7 (0.03) |
| 2015-2018 | 29.5 (0.2) | 28.0 (0.03) |
| BMI in the NHANES cohort was determined using standardized height and weight measurements during the Mobile Examination Center visit. BMI in the NHIS cohort was determined using self-reported height and weight. | | |

| Supplementary Table S5. BMI and All-Cause Mortality Hazard Ratios (95% CI) by Age Group | | | | | | | | |
| --- | --- | --- | --- | --- | --- | --- | --- | --- |
| N = 44,308 |  |  | Overall | | Age 20-64 | | Age 65+ | |
| 1999-2018 NHANES Data | # events/N among overall cohort | # events/N among healthy, never smokers | Overall  (6425/43880) | Healthy, Never Smokers  (1510/20503) | Overall  (1937 deaths/33119) | Healthy, Never Smokers  (467 deaths/17234) | Overall  (4448 deaths/10761) | Healthy, Never Smokers  (1043 deaths/3269) |
| Adjusted HR (95% CI) |  |  |  |  |  |  |  |  |
| <22.5 kg/m2 | 998/6369 | 226/3062 | 1.36 (1.21, 1.52) | 1.28 (1.00, 1.64) | 1.37 (1.11, 1.70) | 1.25 (0.76, 2.04) | 1.36 (1.20, 1.54) | 1.30 (1.00, 1.70) |
| 22.5-24.9 | 1024/6841 | 218/3233 | 1.00 (Ref) | 1.00 (Ref) | 1.00 (Ref) | 1.00 (Ref) | 1.00 (Ref) | 1.00 (Ref) |
| 25-27.5 | 1252/7852 | 293/3598 | 0.97 (0.86, 1.10) | 1.03 (0.80, 1.32) | 0.99 (0.80, 1.24) | 1.22 (0.76, 1.95) | 0.98 (0.87, 1.12) | 0.97 (0.74, 1.28) |
| 27.5-29.9 | 1112/7248 | 281/3314 | 0.89 (0.79, 0.99) | 0.86 (0.67, 1.10) | 0.87 (0.70, 1.08) | 0.90 (0.56, 1.44) | 0.93 (0.83, 1.05) | 0.89 (0.68, 1.16) |
| 30.0-34.9 | 1290/9162 | 322/4229 | 1.01 (0.91, 1.12) | 1.08 (0.87, 1.34) | 1.05 (0.87, 1.28) | 1.38 (0.87, 2.20) | 1.03 (0.93, 1.14) | 1.06 (0.83, 1.35) |
| ≥35 | 881/6836 | 208/3254 | 1.37 (1.22, 1.54) | 1.21 (0.94, 1.56) | 1.61 (1.29, 2.01) | 1.61 (1.07, 2.43) | 1.29 (1.14, 1.47) | 1.15 (0.86, 1.53) |
| Adjusted HR (95% CI) – Additionally adjusted for metabolic syndrome criteria | | | |  |  |  |  |  |
| <22.5 kg/m2 | 998/6369 | 226/3062 | 1.36 (1.21, 1.52) | 1.30 (0.99, 1.69) | 1.41 (1.11, 1.79) | 1.19 (0.70, 2.04) | 1.37 (1.18, 1.60) | 1.31 (1.00, 1.73) |
| 22.5-24.9 | 1024/6841 | 218/3233 | 1.00 (Ref) | 1.00 (Ref) | 1.00 (Ref) | 1.00 (Ref) | 1.00 (Ref) | 1.00 (Ref) |
| 25-27.5 | 1252/7852 | 293/3598 | 0.97 (0.86, 1.10) | 0.98 (0.74, 1.28) | 0.94 (0.73, 1.21) | 1.06 (0.62, 1.79) | 0.96 (0.83, 1.10) | 0.98 (0.72, 1.33) |
| 27.5-29.9 | 1112/7248 | 281/3314 | 0.89 (0.79, 0.99) | 0.76 (0.57, 1.00) | 0.77 (0.60, 0.98) | 0.71 (0.41, 1.24) | 0.87 (0.75, 1.01) | 0.83 (0.62, 1.12) |
| 30.0-34.9 | 1290/9162 | 322/4229 | 1.01 (0.91, 1.12) | 0.92 (0.73, 1.17) | 0.90 (0.72, 1.12) | 1.04 (0.62, 1.76) | 0.98 (0.87, 1.10) | 0.98 (0.74, 1.29) |
| ≥35 | 881/6836 | 208/3254 | 1.37 (1.22, 1.54) | 1.04 (0.76, 1.41) | 1.30 (1.02, 1.67) | 1.12 (0.70, 1.83) | 1.21 (1.03, 1.41) | 1.07 (0.76, 1.51) |
| Disease definition per Gonzalez et al.: History of non-skin cancer (except melanoma) or cardiovascular disease | | | | | | | | |

|  |  | | Supplementary Table S6. Association between BMI and All-cause Mortality Among NHANES Participants by Waist Circumference | | | | | | | | |
| --- | --- | --- | --- | --- | --- | --- | --- | --- | --- | --- | --- |
| N = 44,308 | |  | |  | | Overall | | Healthy, Never Smokers | | Ever Smokers w/ Disease | |
| 1999-2018 NHANES Data | | N | | High Waist Circumference | N | | Normal Waist Circumference | High Waist Circumference | Normal Waist Circumference | High Waist Circumference | Normal Waist Circumference |
| Adjusted HR (95% CI) | |  | |  |  | |  |  |  |  |  |
| <22.5 kg/m2 | | 33/135 | | 1.50 (0.92, 2.47) | 888/6052 | | 1.39 (1.22, 1.59) | 1.25 (0.95, 1.65) | 0.98 (0.41, 2.38) | 1.78 (0.83, 3.84) | 1.52 (1.17, 1.97) |
| 22.5-24.9 | | 228/961 | | 1.19 (0.99, 1.43) | 736/5689 | | 1.00 (Ref) | 1.07 (0.72, 1.58) | 1.00 (Ref) | 1.37 (0.95, 1.97) | 1.00 (Ref) |
| 25-27.4 | | 626/2946 | | 1.09 (0.94, 1.26) | 558/4695 | | 0.92 (0.79, 1.08) | 1.09 (0.80, 1.49) | 0.96 (0.68, 1.35) | 1.07 (0.83, 1.38) | 0.78 (0.95, 1.97) |
| 27.5-29.9 | | 883/4806 | | 0.96 (0.85, 1.08) | 164/2231 | | 0.69 (0.54, 0.87) | 0.87 (0.66, 1.15) | 0.80 (0.51, 1.27) | 1.00 (0.81, 1.24) | 0.64 (0.38, 1.09) |
| 30-34.9 | | 1193/8330 | | 1.04 (0.93, 1.18) | 16/558 | | 0.64 (0.34, 1.18) | 1.07 (0.82, 1.39) | 1.15 (0.42, 3.17) | 1.06 (0.84, 1.33) | 0.19 (0.03, 1.33) |
| ≥35 | | 881/6836 | | 1.46 (1.28, 1.67) | | | | 1.26 (0.95, 1.68) | | 1.34 (1.06, 1.69) | |
| High waist circumference defined as ≥102 cm for men and ≥88 cm for women. Normal waist circumference is <102 cm for men and <88 for women. Sample size was too small to calculate hazard ratios for Class II+ obese participants with normal waist circumference so they are grouped together. | | | | | | | | | | | |

| Supplemental Table S7. Association between BMI and All-cause Mortality by Weight Change Status | | | | | | | | | | |
| --- | --- | --- | --- | --- | --- | --- | --- | --- | --- | --- |
| N = 44,308 | Overall | | | | | | | Healthy, Never Smokers (N=19,940) | | |
| 1999-2018 NHANES Data | N | Unintentional Weight Loss | N | Intentional Weight loss / No weight change | N | Weight Gain | Unintentional Weight Los | | Intentional Weight loss / No weight change | Weight Gain |
| Adjusted HR (95% CI) |  |  |  |  |  |  |  | |  |  |
| <22.5 kg/m2 | 205/676 | 2.44 (1.97, 3.03) | 721/5049 | 1.33 (1.17, 1.52) | 52/540 | 2.43 (1.72, 3.43) | 2.77 (1.85, 4.15) | | 1.23 (0.95, 1.59) | 1.68 (0.64, 4.43) |
| 22.5-24.9 | 143/552 | 1.97 (1.55, 2.50) | 772/5280 | 1.00 (Ref) | 85/886 | 1.27 (0.97, 1.66) | 1.73 (0.98, 3.05) | | 1.00 (Ref) | 0.83 (0.38, 1.81) |
| 25-27.4 | 138/1177 | 1.59 (1.25, 2.03) | 970/5880 | 0.99 (0.86, 1.14) | 120/1236 | 1.48 (1.20, 1.82) | 1.84 (1.01, 3.35) | | 1.02 (0.78, 1.32) | 1.54 (0.95, 2.48) |
| 27.5-29.9 | 108/486 | 1.57 (1.19, 2.08) | 817/5257 | 0.90 (0.80, 1.02) | 151/1328 | 1.14 (0.90, 1.43) | 1.29 (0.68, 2.44) | | 0.80 (0.61, 1.05) | 1.01 (0.62, 1.65) |
| 30-34.9 | 122/564 | 1.80 (1.42, 2.28) | 890/6117 | 1.01 (0.91, 1.13) | 243/2242 | 1.28 (1.03, 1.57) | 1.32 (0.75, 2.30) | | 1.05 (0.83, 1.33) | 1.32 (0.84, 2.09) |
| ≥35 | 65/353 | 2.29 (1.64, 3.20) | 575/4159 | 1.43 (1.24, 1.65) | 208/2058 | 1.68 (1.38, 2.05) | 3.03 (1.46, 6.29) | | 1.21 (0.90, 1.62) | 1.36 (0.90, 2.04) |
| Unintentional weight loss defined as reporting unintentionally losing >10 pounds in the past year. Intentional weight loss defined as losing >10 pounds intentionally. No weight change defined as losing or gaining <10 pounds in the past year. Weight gain defined as >10 pound weight gain in the past year. All weight changes based on self-report. | | | | | | | | | | |

| Supplemental Table S8. Association between BMI and Mortality Among NHANES Participants by Different Disease Definitions | | | | | | |
| --- | --- | --- | --- | --- | --- | --- |
|  | Disease-free per Gonzalez et al. | | Disease-free per Calle et al. | | Comprehensive Disease-free Definition | |
| 1999-2018 NHANES Data | Overall  (3488/35345) | Never Smokers  (1458/19940) | Overall  (2542 deaths/28932) | Never Smokers  (1131 deaths/16997) | Overall  (1892 deaths/27560) | Never Smokers  (817 deaths/16527) |
| Adjusted HR (95% CI) |  |  |  |  |  |  |
| <22.5 kg/m2 | 1.36 (1.18, 1.58) | 1.31 (1.02, 1.68) | 1.27 (1.06, 1.53) | 1.11 (0.84, 1.47) | 1.26 (1.01, 1.58) | 1.14 (0.83, 1.58) |
| 22.5-24.9 | 1.00 (Ref) | 1.00 (Ref) | 1.00 (Ref) | 1.00 (Ref) | 1.00 (Ref) | 1.00 (Ref) |
| 25-27.5 | 0.97 (0.82, 1.15) | 1.04 (0.81, 1.34) | 1.04 (0.86, 1.26) | 1.05 (0.81, 1.38) | 1.02 (0.81, 1.29) | 1.13 (0.83, 1.54) |
| 27.5-29.9 | 0.82 (0.71, 0.95) | 0.82 (0.64, 1.05) | 0.88 (0.74, 1.04) | 0.85 (0.65, 1.11) | 0.97 (0.78, 1.19) | 0.98 (0.73, 1.30) |
| 30.0-34.9 | 1.00 (0.88, 1.13) | 1.07 (0.85, 1.33) | 1.05 (0.90, 1.24) | 1.13 (0.88, 1.44) | 1.08 (0.90, 1.30) | 1.26 (0.95, 1.68) |
| ≥35 | 1.33 (1.14, 1.55) | 1.24 (0.96, 1.60) | 1.43 (1.19, 1.72) | 1.38 (1.04, 1.83) | 1.48 (1.19, 1.83) | 1.59 (1.19, 2.13) |
| Disease definition per Gonzalez et al.: History of non-skin cancer (except melanoma) or cardiovascular disease  Disease definition adapted from Calle et al.: History of cancer except nonmelanoma skin cancer, heart disease, stroke, COPD, asthma, self-rated poor health, or unintentional weight loss of at least 10 pounds in the past year  Comprehensive disease definition: Presence of any one of the following: self-reported COPD, non-skin cancer, current liver disease, cardiovascular disease (CAD, HF, stroke, MI), chronic kidney disease (eGFR<60), other kidney disease, asthma, chronic prescription medication use (chronic CNS agents, chronic respiratory agents, anticoagulation, antineoplastic therapy), moderate to severe anemia (Hgb<10 g/dl), malnutrition (serum albumin<3.2 g/dl), unintentional weight loss of ≥10 pounds in the past year.  Disease-free is considered absence of any disease condition. | | | | | | |

| Supplementary Table S9. Association between Maximum Lifetime BMI and Mortality Among NHANES Participants by Age Group | | | | | | |
| --- | --- | --- | --- | --- | --- | --- |
|  | Overall | | Age 20-64 | | Age 65+ | |
| 1999-2018 NHANES Data | Overall  (4424/39659) | Healthy, Never Smokers  (994/18969) | Overall  (1872 deaths/32070) | Healthy, Never Smokers  (450 deaths/16631) | Overall  (2552 deaths/7589) | Healthy, Never Smokers  (544 deaths/2338) |
| Adjusted HR (95% CI) |  |  |  |  |  |  |
| <22.5 kg/m2 | 1.10 (0.89, 1.36) | 1.09 (0.73, 1.63) | 1.18 (0.86, 1.62) | 1.59 (0.84, 3.03) | 1.07 (0.82, 1.39) | 0.78 (0.40, 1.51) |
| 22.5-24.9 | 1.00 (Ref) | 1.00 (Ref) | 1.00 (Ref) | 1.00 (Ref) | 1.00 (Ref) | 1.00 (Ref) |
| 25-27.5 | 0.88 (0.76, 1.01) | 0.83 (0.61, 1.15) | 0.92 (0.71, 1.19) | 1.10 (0.63, 1.92) | 0.87 (0.71, 1.06) | 0.66 (0.45, 0.97) |
| 27.5-29.9 | 0.96 (0.82, 1.13) | 0.87 (0.60, 1.25) | 0.92 (0.71, 1.20) | 1.10 (0.61, 1.99) | 1.03 (0.84, 1.26) | 0.72 (0.52, 1.00) |
| 30.0-34.9 | 1.02 (0.89, 1.17) | 0.97 (0.70, 1.33) | 1.03 (0.83, 1.29) | 1.31 (0.75, 2.31) | 1.05 (0.88, 1.26) | 0.75 (0.52, 1.07) |
| ≥35 | 1.55 (1.34, 1.79) | 1.43 (1.00, 2.05) | 1.57 (1.24, 1.99) | 2.00 (1.15, 3.46) | 1.62 (1.33, 1.98) | 1.07 (0.72, 1.61) |
| N=39,659 (participants without data on maximum lifetime BMI excluded)  Disease definition per Gonzalez et al.: History of non-skin cancer (except melanoma) or cardiovascular disease (Healthy defined as absence of disease)  Because follow-up time was determined via self-reported age at maximum BMI whose answer field grouped all individuals 80+, we restricted this analysis to NHANES participants < 80. | | | | | | |

| Supplementary Table S10. Association between BMI and Mortality Among NHIS: Exclusion of deaths in the first 5 years of follow-up | | |
| --- | --- | --- |
|  |  | |
| 1999-2018 NHIS Data | Overall | Healthy, Never Smokers |
| Adjusted HR (95% CI) |  |  |
| <18.5 kg/m2 | 1.73 (1.57, 1.91) | 1.53 (1.27, 1.84) |
| 18.5-19.9 | 1.32 (1.23, 1.41) | 1.27 (1.13, 1.42) |
| 20.0-22.4 | 1.13 (1.08, 1.18) | 1.06 (0.97, 1.15) |
| 22.5-24.9 | 1.00 (Ref) | 1.00 (Ref) |
| 25.0-27.4 | 1.00 (0.97, 1.04) | 0.99 (0.93, 1.07) |
| 27.5-29.9 | 0.99 (0.96, 1.04) | 1.05 (0.97, 1.13) |
| 30.0-34.9 | 1.14 (1.10, 1.19) | 1.29 (1.20, 1.38) |
| 35.0-39.9 | 1.38 (1.31, 1.45) | 1.56 (1.40, 1.73) |
| ≥40 | 1.86 (1.75, 1.98) | 2.20 (1.94, 2.49) |
| HR: Hazard Ratio. CI: Confidence Interval. Cox models were adjusted for age, gender, race/ethnicity, education, marital status, physical activity, alcohol consumption, insurance coverage, region of residence, and citizenship status  Disease definition per Gonzalez et al.: History of non-skin cancer (except melanoma) or cardiovascular disease (Healthy defined as absence of disease) | | |
