# Supplementary material for: Body mass index and all-cause mortality in a 21st century U.S. population: A National Health Interview Survey analysis
Source: PLoS One. 2023 Jul 5;18(7):e0287218. doi: 10.1371/journal.pone.0287218 (PMC10321632; doi:10.1371/journal.pone.0287218)
Supplement: Attachment — Submitted filename: Plos One review.docx [file pone.0287218.s003.docx]

Thank you for the opportunity to review “Body Mass Index and All-cause Mortality in a 21st Century U.S. Population: A National Health Interview Survey Analysis”. I commend the authors on their study in using representative data to elucidate the associations between BMI and mortality. This study provides a much-needed update to the greater scientific communities understanding of BMI and mortality. Below are my comments, questions, and suggestions.

**Overall**

The proposed novelty of the study is the use of a more contemporary sample of the U.S. population compared to what has been previously seen in the literature. Authors state that “nearly all U.S. studies to date used data from the 1960s through the 1990s and have included predominantly non-Hispanic White men and women.” I think that this is generally true. However, there are some studies utilizing more contemporary national surveillance data sets that also examine hazard ratios specific to race-ethnic groups (see references below). I suggest reframing the introduction to account for these three studies while also simultaneously highlighting how you study accounts for limitations of them (e.g. Linked mortality data through 2019, subgroup analyses by gender and race-ethnicity, use of a more liberal categorization of BMI to examine a more precise dose-response relationship) In other words, I believe your study does a superb job of filling in some large gaps in the literature but does not highlight its strengths as much as it should.

Nguyen et al. Characterising the relationships between physiological indicators and all-cause mortality (NHANES): a population-based cohort study. Lancet Healthy Longev 2021; 2: e651–62. Study uses data from the 1999-2014 NHANES

Zheng et al. The Body Mass Index-Mortality Link across the Life Course: Two Selection Biases and Their Effects. Uses 1999-2010 NHANES

Howell et al. Maximum Lifetime Body Mass Index and Mortality in Mexican American Adults: the National Health and Nutrition Examination Survey III (1988–1994) and NHANES 1999–2010. Uses 199-2010 NHANES data

Suggest changing subjects to particpants

**Abstract**

No suggestions

**Introduction**

See “overall” comments. The addition of the three suggested studies to your references highlights that, yes there are some contemporary population-based studies that attempt to answer this question but still have limitations that your study circumvents. A couple of sentences discussing theses studies and their limitations would strengthen your rationale.

**Methods**

Were the STROBE guidelines followed for this study?

Suggest providing the references that detail the linking methodology for the NDI to the NHIS.

There are some studies that suggest the use of two years for mortality during follow-up exclusion may not be enough to control for residual confounding. Did you perform any sensitivity analysis with a larger follow-up time?

5 years exclusion criteria: Di Angelantonio E, Bhupathiraju SN, Wormser D, Gao P, Kaptoge S, De Gonzalez AB, Cairns BJ, Huxley R, Jackson CL, Joshy G, Lewington S. Body-mass index and all-cause mortality: individual-participant-data meta-analysis of 239 prospective studies in four continents. The Lancet. 2016 Aug 20;388(10046):776-86.

I’m wondering if having the NHANES analysis as a secondary, sensitivity analysis diminishes its potential impact. Why not include it in your abstract and primary methods? Other studies have done this. The results from your NHANES analysis are quite impactful. It is possible that it is a space issue. Nonetheless, the NHANES analysis provides good results.

I understand the rationale for not adjusting for comorbidities in the main analysis but why not conduct subgroup analysis by disease status (e.g. CVD, cancer, diabetes, hypertension) similar to Calle et al.? It seems you might have done this in your results for diabetes and hypertension (See first comment under **results** heading) but there is nothing written about it. It might be prudent to see if the dose-response seen in the overall sample holds when you restrict analysis to those with disease.

Calle EE, Thun MJ, Petrelli JM, Rodriguez C, Heath Jr CW. Body-mass index and mortality in a prospective cohort of US adults. New England Journal of Medicine. 1999 Oct 7;341(15):1097-105

**Results**

Page six, third paragraph seems to be missing results. “Upon additionally controlling for comorbidities including diabetes and hypertension,” there are no results listed after this.

**Discussion**

No suggestions
