# Supplementary material for: Body mass index and all-cause mortality in a 21st century U.S. population: A National Health Interview Survey analysis
Source: PLoS One. 2023 Jul 5;18(7):e0287218. doi: 10.1371/journal.pone.0287218 (PMC10321632; doi:10.1371/journal.pone.0287218)
Supplement: Attachment — Submitted filename: BMI_mortality_PLOSOne_ResponsetoReviewer.docx [file pone.0287218.s004.docx]

Response to Reviewers

PONE-D-22-25310

January 30, 2023

Dear Dr. Ehrlich,

Thank you for your comments and suggestions regarding our study (ID: PONE-D-22-25310) titled, “*Body Mass Index and All-cause Mortality in a 21st Century U.S. Population: A National Health Interview Survey Analysis*”. We have addressed all concerns and believe the comments have improved our manuscript. Below are the corrections/responses to each of the remarks. Changes to the manuscript text are also marked in bold in the manuscript file.

**Responses to Reviewer #1**

**1. The proposed novelty of the study is the use of a more contemporary sample of the U.S. population compared to what has been previously seen in the literature. Authors state that “nearly all U.S. studies to date used data from the 1960s through the 1990s and have included predominantly non-Hispanic White men and women.” I think that this is generally true. However, there are some studies utilizing more contemporary national surveillance data sets that also examine hazard ratios specific to race-ethnic groups (see references below). I suggest reframing the introduction to account for these three studies while also simultaneously highlighting how you study accounts for limitations of them (e.g. Linked mortality data through 2019, subgroup analyses by gender and race-ethnicity, use of a more liberal categorization of BMI to examine a more precise dose-response relationship) In other words, I believe your study does a superb job of filling in some large gaps in the literature but does not highlight its strengths as much as it should.**

Thank you for your thorough review of our manuscript. It was much appreciated by our research team. We have now reframed the introduction to better address newer studies that have utilized data from the 2000-2010s, including the ones you shared. As you mentioned, some strengths of our study beyond the more recent study period include 1) subgroup analyses with sufficient power given our large sample size. NHANES data, while rich in clinical information, is far smaller than the NHIS data used in our primary analysis, and 2) comprehensive adjustment for bias and confirmation of findings in both NHIS and NHANES datasets.

Revision:

“*Of the few studies using more contemporary populations^15-87^, they were restricted by small sample sizes limiting analysis in racial and gender subgroups, had inadequate adjustment for methodologic bias including reverse causality, collider bias, effect modification, and the healthy person effect, and/or only rudimentarily characterized the dose-response relationship between BMI and mortality.*” (Introduction, Paragraph 2, Page 3)

**2. Suggest changing subjects to particpants**

Thank you for this suggestion. We have now changed the term ‘subjects’ to ‘participants’ throughout the manuscript text.

**3. Introduction: See “overall” comments. The addition of the three suggested studies to your references highlights that, yes there are some contemporary population-based studies that attempt to answer this question but still have limitations that your study circumvents. A couple of sentences discussing theses studies and their limitations would strengthen your rationale.**

Thank you again for the suggestion. As noted above, we have changed the introduction to reflect the three studies suggested as well as one other study we found upon literature review.

**4. Were the STROBE guidelines followed for this study?**

Yes, we made sure to use the STROBE guidelines checklist while preparing the manuscript.

**5. Suggest providing the references that detail the linking methodology for the NDI to the NHIS.**

Thank you for this suggestion. We have now included the citation for the analytic file detailing the linking methodology for NDI to any National Center for Health Statistics (NCHS) survey.

Citation: National Center for Health Statistics. The Linkage of National Center for Health Statistics Survey Data to the National Death Index — 2019 Linked Mortality File (LMF): Linkage Methodology and Analytic Considerations, June 2022. Hyattsville, Maryland. Available at the following address: https://www.cdc.gov/nchs/data-linkage/mortality-methods.htm.

**6. There are some studies that suggest the use of two years for mortality during follow-up exclusion may not be enough to control for residual confounding. Did you perform any sensitivity analysis with a larger follow-up time?**

Thanks for this insightful comment. We did conduct several sensitivity analyses to account for reverse causality and residual confounding, including increasing the follow-up exclusion to 5 years as was done in the meta-analysis referenced. Exclusion of early deaths is a technique to try and account for subclinical/undiagnosed disease that may lead to weight loss and thus disproportionately contribute to lower BMI groups. However, we did not find any significant change in risk of mortality across any of the BMI categories. Several studies have noted similar findings – that increasing the exclusion follow-up time period may not significantly impact risks; however, it can lead to significant selection bias because a sizeable portion of the population can be excluded. We thus opted not to exclude 5 years of follow-up. To clarify this further, we explained this in more detail in the discussion and also included a **supplementary table S10** that excludes the first 5 years of deaths.

Revisions:

*“To examine the potential impact of reverse causality due to subclinical disease, we conducted analyses limited to participants who did not die within the first two years of follow-up. Although some prior meta-analyses have excluded the first five years of follow-up, there has been concern that such exclusions would lead to significant selection bias as a sizeable proportion of the study population would be excluded, without significant benefit in reducing reverse causality. Nevertheless, we conducted sensitivity analyses excluding the first five years of follow-up.”* (Methods, Statistical Analysis, Paragraph 3, pg. 5)

See Supplementary Table S10.

**7. I’m wondering if having the NHANES analysis as a secondary, sensitivity analysis diminishes its potential impact. Why not include it in your abstract and primary methods? Other studies have done this. The results from your NHANES analysis are quite impactful. It is possible that it is a space issue. Nonetheless, the NHANES analysis provides good results.**

Thank you for this comment. We agree with your assessment that the NHANES results are quite impactful and provide further insight complementary to NHIS findings. However, due to word count/table limitations, we could not include it in the primary analysis and is instead in the supplement. We felt the NHIS, given its large sample size, provided much more robust estimates and allowed for subgroup analyses so it was used as the primary analysis.

**8. I understand the rationale for not adjusting for comorbidities in the main analysis but why not conduct subgroup analysis by disease status (e.g. CVD, cancer, diabetes, hypertension) similar to Calle et al.? It seems you might have done this in your results for diabetes and hypertension (See first comment under results heading) but there is nothing written about it. It might be prudent to see if the dose-response seen in the overall sample holds when you restrict analysis to those with disease.**

Thank you for this comment. We in fact did do subgroup analyses by disease status as determined by Calle et al., Berrington de Gonzalez et al., and our own more rigorous definition of disease. We acknowledge that this was likely not clear in the manuscript, so we have clarified this now in the revised manuscript. All figures in the main analysis have a subgroup of healthy, never-smokers who do not have CVD or cancer. In the supplementary file, Figure S2 shows the BMI-mortality associations by disease-free status. The red line depicts never-smoking individuals without minor morbidity, defined as self-reported history of cardiovascular disease, non-skin cancer except melanoma, COPD, current asthma, liver disease, kidney disease, diabetes, or functional limitations. The blue line depicts never-smoking participants without major morbidity, defined via Berrington de Gonzalez et al. as self-reported history of cardiovascular disease and non-skin cancer except melanoma. We did not show individuals with disease because of the aforementioned biases of reverse causality and confounding by pre-existing disease. Although we do not show the data, we did conduct the analysis which showed the BMI-mortality curve shifts downwards, suggesting more inverse associations with mortality at overweight BMI ranges. However, this is more likely to be due to confounding than a true inverse association.

Revision:

*“We further conducted analyses among subgroups of disease-free individuals, utilizing different definitions of disease: minor morbidity, defined as self-reported history of cardiovascular disease, non-skin cancer except melanoma, COPD, current asthma, liver disease, kidney disease, diabetes, or functional limitations; and major morbidity, defined via Berrington de Gonzalez et al.4 as self-reported history of cardiovascular disease and non-skin cancer except melanoma.”* (Methods, Statistical Analysis, Paragraph 2, Pg. 4)

**9. Results: Page six, third paragraph seems to be missing results. “Upon additionally controlling for comorbidities including diabetes and hypertension,” there are no results listed after this.**

Thank you for catching this typo. We had initially intended on deleting the sentence completely. However, we have now updated the text to complete the sentence to illustrate the concept of collider bias. This was meant to be more of a sensitivity analysis as adjustment for obesity-related comorbidities such as diabetes and hypertension would likely lead to collider bias, leading to the appearance that mortality risks are lower than they really are.

Revision:

*“Upon additionally controlling for comorbidities including diabetes and hypertension (potential colliders), risk of mortality marginally decreased among overweight BMI categories (BMI 20.0-22.4: 1.10 [1.03,1.19], BMI 22.5-24.9: 1.00 [Ref], BMI 25.0-27.4: 0.95 [0.89,1.00], BMI 27.5-29.9: 0.96 [0.90, 1.02]).”* (Results, Paragraph 2, Page 6)

**Responses to Reviewer #2**

**10. Page 5: Covariates. I understand the need for the supplement, but I recommend that a bit more detail be added on the covariates. Just mentioning them would be fine knowing that readers can seek out the supplement to learn about the details of specific categories.**

Thank you for this suggestion. We had included the list of covariates that we adjusted for in the Cox proportional hazards regression in the ‘Statistical Analysis’ section but realized that they were not explicitly mentioned in the Covariate subsection. We have now moved the text from the Statistical Analysis section to the Covariates subsection and provided more detail.

*“Cox models were adjusted for age, gender, race/ethnicity, education, marital status, physical activity, alcohol consumption, insurance coverage, region of residence, and citizenship status.”* (Methods, Statistical Analysis, Paragraph 1, pg. 4)

**11. 88 Page 5: Statistical Procedures. Please provide more detail on the analyses and procedures that were used within SAS.**

Thank you for this comment. We have now provided further information on the specific SAS analyses used.

**Responses to Academic Editor Comments**

**12. Thank you for this well written submission. I suggest that the Abstract should mention that the analyses were cox proportional hazards models, and agree with the Reviewer's comment that more info on the selected covariates would be appropriate. I look forward to your revision and response to the Reviewers' comments.**

Thank you for your comment. We have now included Cox proportional hazards modeling in the abstract methods text. We have also reformatted and condensed the abstract text to fit the 300 word limit and to provide more methodological detail.

*“We estimated risk of all-cause mortality using multivariable Cox proportional hazards regression, adjusting for covariates, accounting for the survey design, and performing subgroup analyses to reduce analytic bias*” (Abstract, Methods, Page 2)

**Editorial Comments:**

1. Please ensure that your manuscript meets PLOS ONE's style requirements, including those for file naming.

**Confirmed.**

2. Please provide additional details regarding participant consent. In the ethics statement in the Methods and online submission information, please ensure that you have specified (1) whether consent was informed and (2) what type you obtained (for instance, written or verbal, and if verbal, how it was documented and witnessed). If your study included minors, state whether you obtained consent from parents or guardians. If the need for consent was waived by the ethics committee, please include this information.

**This is now clarified in the text. This was a retrospective study of deidentified, publicly available data that is not considered human subjects research; thus no consent or IRB was needed.**

3. Please include your tables as part of your main manuscript and remove the individual files. Please note that supplementary tables (should remain/ be uploaded) as separate "supporting information" files

**Done.**

4. Please include captions for your Supporting Information files at the end of your manuscript, and update any in-text citations to match accordingly. Please see our Supporting Information guidelines for more information: http://journals.plos.org/plosone/s/supporting-information.

**Confirmed.**

5. Please review your reference list to ensure that it is complete and correct. If you have cited papers that have been retracted, please include the rationale for doing so in the manuscript text, or remove these references and replace them with relevant current references. Any changes to the reference list should be mentioned in the rebuttal letter that accompanies your revised manuscript. If you need to cite a retracted article, indicate the article’s retracted status in the References list and also include a citation and full reference for the retraction notice.

**Done.**
