# Supplementary material for: Body mass index and all-cause mortality in a 21st century U.S. population: A National Health Interview Survey analysis
Source: PLoS One. 2023 Jul 5;18(7):e0287218. doi: 10.1371/journal.pone.0287218 (PMC10321632; doi:10.1371/journal.pone.0287218)
Supplement: Attachment — Submitted filename: BMI_mortality_PLOSOne_ResponsetoReviewer_5.28.docx [file pone.0287218.s005.docx]

Response to Reviewers

PONE-D-22-25310

May 28, 2023

Dear Dr. Ehrlich,

Thank you for your additional comments and suggestions regarding our study (ID: PONE-D-22-25310) titled, “*Body Mass Index and All-cause Mortality in a 21st Century U.S. Population: A National Health Interview Survey Analysis*”. We have addressed all concerns and believe the comments have improved our manuscript. Below are the corrections/responses to each of the remarks. Changes to the manuscript text are also marked as tracked changes in the manuscript file.

**Responses to Reviewer #1**

**1. Please add mention that the STRBE guidelines checklist was followed in preparing this manuscript.**

Thank you for this suggestion. We have now included a sentence in the Methods section confirming we have used the STROBE guidelines checklist.

**2. In the Introduction, statements pertaining to racial diversity and racial subgroups (e.g., ‘more racially diverse’) should also include ‘ethnicity’ (e.g., ‘more racially and ethnically diverse’).**

Thank you for pointing out this oversight. We have now included ‘ethnicity’ in the mentioned sentence.

Revision: “In contrast, the contemporary U.S. population: (1) has a substantially different BMI distribution, with mean BMI having risen by more than 2 kg/m2 in both men and women since the 1970s, and increasing skewness towards obese-range BMIs; (2) has seen >10 year increases in life expectancy both overall and among obese individuals; (3) *is more racially and ethnically diverse…*” (Introduction, Page 3)

**3. There is a statement in the Introduction, ‘inadequate adjustment for methodological bias including reverse causality, collider bias, effect modification, and health person effect’. Perhaps this needs to be split into several sentences in order to clarify the points being made. For one, effect modification is not a methodological bias (though an inadequate sample size to investigate effect modification would be a limitation to prior work).**

Thank you for this comment. We have now changed the wording of the sentence and provided more descriptions of each of the sources of bias.

Revision: “Of the few studies using more contemporary populations, they were either restricted by small sample sizes limiting analysis in racial and gender subgroups, and/or only rudimentarily characterized the dose-response relationship between BMI and mortality. *Furthermore, studies inconsistently adjusted for methodologic bias including confounding by illness-related weight loss, collider bias (e.g. the concept that conditioning on obesity-related disease may distort associations between risk factors like BMI and diet and subsequently bias downstream associations), and the healthy person effect (e.g. selection bias that may be introduced when selecting participants of different BMI groups who may otherwise be healthy).”* (Introduction, Page 3)

**4. For the covariates paragraph (i.e., just prior to the ‘Statistical Analysis’ section): I suggest rephrasing ‘non-skin cancer except melanoma’ for clarity (here and throughout the manuscript, I believe this adjustment includes cancers at various sites but only melanoma for skin cancer). I also suggest including clarification of the ‘healthcare utilization factor’ variable (in parenthesis, as is done for the other covariates). Please indicate the specific supplement section where the covariate categorization is provided (and throughout the manuscript, for all mentions of ‘provided in the supplement’, please state a specific supplement section).**

Thank you for this comment. We have clarified the phrasing of the cancer comorbidity covariate to read as “non-skin cancer or melanoma”. We have also included a description of the healthcare utilization factors included in our analysis, and specified the specific supplement section to be referenced.

Revision: “…comorbidities (self-reported history of cardiovascular disease, non-skin cancer or melanoma, COPD, current asthma, liver disease, kidney disease, diabetes, or functional limitations), and healthcare utilization factors (doctor’s visit in the past 12 months, mental health visit in the past 12 months). Covariate categorizations are provided in the supplement (Extended Supplementary Methods)” (Methods, BMI, Mortality, and Covariates, Page 4)

**5. In the Statistical Analysis section, ‘we did not adjust for comorbidities in the main analysis, as they could be on the causal pathway between BMI and mortality or potential colliders’: Without including a DAG and explanation of potential intermediate variables at play, it is difficult to follow the potential collider argument. BMI may be causally associated with a comorbidity, such as diabetes (i.e., an arrow from BMI leading to diabetes), and diabetes causally associated with mortality (i.e., an arrow from diabetes to mortality, the causal pathway argument). However, I find it hard to justify an arrow stemming from mortality to diabetes, indicating that mortality is ‘causing’ diabetes, which would be the case if diabetes were hypothesized to be a collider here. I suggest clarifying with a DAG or removing all reference to collider bias.**

Thank you for this thoughtful comment. We have now included a DAG in the Supplement (Supplementary Figure S1) to illustrate the ways in which intermediary conditions such as diabetes and hypertension may be colliders. Briefly, we can posit that BMI is causally associated with diabetes and diabetes is causally associated with mortality. If there is an unmeasured confounder, such as poor nutrition, that is associated with BMI, and is causally associated with diabetes and mortality, diabetes becomes a collider (BMI and poor nutrition ‘collide’ on diabetes). Adjusting for diabetes but not for diet (as it is unmeasured) may create a spurious association between BMI and diet and affect the association between BMI and mortality through this pathway. Preston and Stokes explain this phenomenon in much greater detail and more eloquently in the following paper: Preston SH, Stokes A. Obesity paradox: conditioning on disease enhances biases in estimating the mortality risks of obesity. Epidemiology (Cambridge, Mass.). 2014 May;25(3):454.

Revision: “Consistent with prior studies, we did not adjust for comorbidities in the main analysis, as they could be on the causal pathway between BMI and mortality or potential colliders for BMI and unmeasured confounders^3-5^ (Supplementary Figure S1).”

**Supplementary Figure S1.** Directed Acyclic Graph on BMI and All-cause Mortality

Figure caption: The following figure is a crude representation of our proposed DAG for the association between BMI and all-cause mortality. We can assume BMI is causally associated with diabetes and diabetes is causally associated with mortality. If there is an unmeasured confounder, such as poor nutrition, that is associated with BMI, and is causally associated with diabetes and mortality, diabetes becomes a collider (BMI and poor nutrition ‘collide’ on diabetes). Adjusting for diabetes but not for diet (as it is unmeasured) may create a spurious association between BMI and diet and affect the association between BMI and mortality through this pathway. Preston and Stokes explain this phenomenon in much greater detail and more eloquently in the following paper: Preston SH, Stokes A. Obesity paradox: conditioning on disease enhances biases in estimating the mortality risks of obesity. Epidemiology (Cambridge, Mass.). 2014 May;25(3):454.

**6. Also in the Statistical Analysis section: ‘To examine the potential impact of reverse causality due to subclinical disease…’, I suggest including mention/brief clarification of what is meant by the term ‘reverse causality’, as eloquently described by your reference, Banack et al. 2019:**

**‘in the context of obesity-mortality research, the term reverse causality is often used to refer to a situation in which disease status affects both exposure and outcome, because disease often causes weight loss and disease increases mortality risk. Despite being called reverse causality, this is actually a concept that fits the standard definition of confounding in epidemiology…. This is why, in the context of obesity-mortality studies, the phrase reverse causality is often used interchangeably with the terms “confounding by preexisting disease” or “confounding by illness-related weight loss”**

**The readership of PLOS One includes epidemiologists, obesity-mortality researchers, and others, and without clarification, the language may be confusing to some.**

Thank you for this clarification. We have now changed or supplemented all reference to ‘reverse causality’ with ‘confounding by illness-related weight loss’ which we feel is definitely more intuitive to understand and can be appreciated by all readers.

Revision: “To examine the potential impact of *reverse causality (i.e. confounding by illness-related weight loss)*, we conducted analyses limited to participants who did not die within the first two years of follow-up.” (Methods, Statistical Analysis, Page 5)

Revision: “Furthermore, studies inconsistently adjusted for methodologic bias including *confounding by illness-related weight loss*, collider bias…” (Introduction, Page 3)

**7. In the Results section, characteristics: ‘Between the first four-year cycle (1999-2002) and the last cycle (2015-2018), mean BMI rose from 26.7 to 28.0 kg/m2 and prevalence of BMI of ≥30 increased from 22% to 31% (p<0.001 for trend; Supplementary Figure S1). Nearly 21% had BMI of 25.0-27.4, and 14% had BMI of 27.5-29.9.’ Supplementary Table S1 displays Mortality Rates by Subgroups, and Supplementary Table S2 displays Mortality Rates by Survey Cycle Year. Neither of these tables presents prevalence estimates directly (though these can be calculated from the data included in Table 2) so I wonder if the Supplementary Figure S1 reference here was in error? Could you clarify whether, ‘Nearly 21% had BMI of 25.0-27.4, and 14% had BMI of 27.5-29.9’ encompasses prevalence estimates for all years combined?**

Thank you for pointing this out. Supplementary Figure S1 (now Supplementary Figure S2) Panel C shows the distribution of BMI from the first four-year cycle to the last. We realize that it is not possible to calculate the mean BMI or the percent increase in BMI>=30 visually from that so we have now removed reference to Figure S1. The statement, “Nearly 21% had BMI of 25.0-27.4, and 14% had BMI of 27.5-29.9’ encompasses prevalence estimates for all years combined” refers to the prevalence for all years combined and is from Table 1.

Revision: “Between the first four-year cycle (1999-2002) and the last cycle (2015-2018), mean BMI rose from 26.7 to 28.0 kg/m^2^ and prevalence of BMI of ≥30 increased from 22% to 31% (p<0.001 for trend; see **Supplementary Figure S2** for distribution of BMI). Nearly 21% had BMI of 25.0-27.4, and 14% had BMI of 27.5-29.9 (**Table 1**).” (Results, Sample Characteristics, Page 5)

**In the Results section, Race/ethnicity: Supplementary Table S1 does not present five-year mortality rates and unadjusted risks by race/ethnicity, please add these data or indicate ‘data not show’ (though I prefer adding the data). Did the difference between Hispanic adults with BMIs from 25.0 to 29.9 vs. other groups attain statistical significance? Was this examined with a cross product?**

Thanks for pointing out this oversight. We had included some data in the supplementary text underneath Supplementary Table S1 that we have now included in the main text: “Among non-Hispanic White, non-Hispanic Black, and Hispanic participants, 5-year mortality was highest in those with BMI <18.5 (White: 40 [95% CI, 38-43] per 1,000 person-years, Black: 43 [37-50], Hispanic: 20 [16-25]). Five-year mortality was lowest at 11.7 (11.2-12.2) among those with BMI of 30.0-34.9 in White adults and 10.0 (8.8-11.3) at BMI of 35.0-39.9 in Black adults, and 7.1 [6.5-7.8] in Hispanic adults.”

The multiplicative interaction (cross-product) between race/ethnicity and BMI on all-cause mortality did not reach significance after adjustment for covariates. However, we felt it was still important to include racial/ethnic subgroups because of the well-studied racial/ethnic differences in various components of body composition and CV risk factors. We have now more explicitly stated that in the text.

Revision: “These patterns persisted, albeit insignificantly, after adjustment (Figure 4, P-interaction = 0.21).” (Results, Race/ethnicity, Page 6)

**8. Discussion section: As you suggest, time period effects (e.g., differences in the racial ethnic make-up of the U.S. population over time) may explain the contrasting results, therefore mention of the time period under study for all prior work would be help to include (i.e., the meta-analysis by Flegal et al. covered what time frame/period?).**

Thank you for this comment. We have now included the time periods for all the referenced studies in the discussion section.

**9. Discussion section: ‘weight change over time can both confound associations with mortality’ is confusing as written, please clarify.**

Apologies for the confusing wording. We have now changed the wording slightly to have it read better.

Revision: “Further, BMI alone may be insufficient in classifying high-risk adiposity – both waist circumference^31,32^ and weight change over time can modify associations BMI-mortality associations, as seen in our NHANES findings” (Discussion, Page 8)

**10. Discussion section: Second to last paragraph, ‘In summary, our study of a contemporary representative….’ includes recommendations and conclusions outside of the scope of the data presented here (e.g., ‘Clinicians patients may benefit from using complementary measures of adiposity…’ and ‘guidance on management of overweight and obesity’). I suggest removing this paragraph and including mention of the limitations of BMI as a measure in the previous paragraph (or elsewhere). The last paragraph does a lovely job of summarizing the main take away points from these data and analyses.**

Thank you for this comment. We have adjusted the final two paragraphs of the discussion to exclude any out-of-scope implications and combined them into one paragraph.

Revision: “In conclusion, our findings suggest that BMI in the overweight range is generally not associated with increased risk of all-cause mortality. Our study suggests that BMI may not necessarily increase mortality independently of other risk factors in those with BMI of 25.0-29.9 and in older adults with BMI of 25.0-34.9. Consequently, this highlights the potential limitations of BMI in capturing true adiposity and limitations of its clinical value independent of traditional metabolic syndrome criteria. Longitudinal studies incorporating weight history, complementary measures of body composition and body fat distribution (e.g. waist circumference, waist-to-hip ratio), undermeasured consequences of weight (e.g. psychological toll of obesity), and morbidity outcomes are needed to fully characterize the relationship between BMI and mortality.” (Discussion, Page 8-9)

Sincerely,

Aayush Visaria
